# The Changing Integrin Expression and a Role for Integrin β8 in the Chondrogenic Differentiation of Mesenchymal Stem Cells

**DOI:** 10.1371/journal.pone.0082035

**Published:** 2013-11-27

**Authors:** Vanessa L. S. LaPointe, Amanda Verpoorte, Molly M. Stevens

**Affiliations:** Departments of Materials and Bioengineering, and the Institute of Biomedical Engineering, Imperial College London, London, United Kingdom; University of Reading, United Kingdom

## Abstract

Many cartilage tissue engineering approaches aim to differentiate human mesenchymal stem cells (hMSCs) into chondrocytes and develop cartilage *in vitro* by targeting cell-matrix interactions. We sought to better inform the design of cartilage tissue engineering scaffolds by understanding how integrin expression changes during chondrogenic differentiation. In three models of *in vitro* chondrogenesis, we studied the temporal change of cartilage phenotype markers and integrin subunits during the differentiation of hMSCs. We found that transcript expression of most subunits was conserved across the chondrogenesis models, but was significantly affected by the time-course of differentiation. In particular, *ITGB8* was up-regulated and its importance in chondrogenesis was further established by a knockdown of integrin β8, which resulted in a non-hyaline cartilage phenotype, with no *COL2A1* expression detected. In conclusion, we performed a systematic study of the temporal changes of integrin expression during chondrogenic differentiation in multiple chondrogenesis models, and revealed a role for integrin β8 in chondrogenesis. This work enhances our understanding of the changing adhesion requirements of hMSCs during chondrogenic differentiation and underlines the importance of integrins in establishing a cartilage phenotype.

## Introduction

Articular hyaline cartilage has a low capacity for self-repair following traumatic injury or degeneration from osteoarthritis, a disease associated with the increasingly ageing population. The extent of endogenous repair is related to the size of the defect and is limited by the avascular nature of cartilage and the low mitotic activity of chondrocytes. For many patients, surgical solutions can reduce pain and swelling and improve joint motility, but the resulting tissue typically resembles fibrocartilage, which is mechanically inferior and less durable than articular cartilage, and tends to be accompanied by an immune response [1]. There is therefore an unquestionable need for regenerative medicine approaches to cartilage repair.

A wide range of tissue engineering strategies have been employed but the paradigm for most involves the combination of cells, a scaffold, and soluble or insoluble cues, many iterations of which have been studied [2]. For the cell source, some success has been achieved using chondrocytes [3], bone marrow-derived mesenchymal stem cells (MSCs) [4,5], adipose-derived stem cells [6], embryonic stem cells [7], and combinations thereof [8]. The use of adult stem cells is inspired by their availability (especially compared to autologous chondrocytes) and their ability to be expanded *in vitro*. As with the cell source, the scaffold design has been a widely studied variable. Over recent years, there have been a number of successful engineering approaches, mimicking many of the native properties of cartilage, in the hope that the cell phenotype will respond accordingly. For example, the spatially varying mechanical properties of cartilage were mimicked using polymers and hydrogels [9], the nano- to micrometre topography was mimicked with polymer fibres [10,11], and the extracellular matrix (ECM) components of cartilage were preserved through a decellularisation technique [12]. Unfortunately, embryogenesis remains the only reliable means of generating native cartilage, and the best option available to surgeons is a selection of polymer fleeces, sponges, and fibrous materials seeded with the patient’s own stem cells for implantation into the defect site. 

The disparity between the promise of tissue engineering and its actual success in replicating chondrogenesis can be explained by many factors. For example, despite many available differentiation protocols, the establishment of phenotypic chondrocytes, capable of producing a cartilage-like ECM, remains a challenge [13]. Indeed, chondrocytes have a tendency to dedifferentiate towards a fibroblastic phenotype *in vitro*, a phenomenon that has been linked to cell-matrix interactions that become disregulated over time. The importance of the ECM as a survival factor has long been known and has also been demonstrated in the case of chondrocytes [14]. Even on surgical implants, providing the correct matrix cues affects chondrocyte phenotype [15]. However, it has been shown that whilst removing chondrocytes from their native ECM leads to dedifferentiation, providing tissue-specific ECM alone is not enough to maintain the phenotype [16]. This may be because the interaction of cells with their ECM is more than for physical attachment, and rather, it importantly provides a source of sequestered growth factors and signalling initiators, all of which are tightly spatially and temporally regulated. 

One of the key families of cell surface receptors involved in cell-ECM interactions is the integrins. They are heterodimeric (one α and one β subunit) transmembrane glycoproteins, consisting of a large globular extracellular domain, capable of specificity to ECM proteins and other cell surface receptors, and a smaller cytoplasmic domain, which interacts with many cytoskeletal proteins and initiates signalling cascades. These interactions are important in chondrocytes and chondrogenesis, as demonstrated in experiments using blocking antibodies. For example, blocking integrin β1 severely interferes with chondrocyte adhesion to fibronectin, type II collagen, and type IV collagen, and inhibits chondrogenesis [17,18]. Integrins are also important in pericellular matrix development and in dedifferentiation, the latter of which can be mitigated by inhibiting the αv or β5 integrin subunits [19,20].

With the importance of cell-ECM and, in particular, integrin-mediated signalling established, we sought to determine the temporal change of integrin expression in different *in vitro* models of chondrogenesis. Many groups use integrin ligands as a means of promoting cell attachment and differentiation, yet it is unknown how integrin expression changes as MSCs become chondrocytes. We therefore aimed to generate the knowledge necessary to improve the design of tissue engineering scaffolds. We found that integrin expression was generally well-conserved across different models of chondrogenic differentiation, but significantly changed during the time-course of differentiation.

## Materials and Methods

### Cell culture

Human bone marrow-derived mesenchymal stem cells (hMSCs) from two donors (one 60 year old female, one 68 year old male) were purchased from PromoCell (UK) (CD44/CD105 > 95% positive, CD31/CD45 > 95% negative). Cells were maintained in growth medium (Mesenchymal Stem Cell Growth Medium; PromoCell, UK) at 37°C and 5% CO_2_. Prior to differentiation experiments, cells were expanded to Passage 4 and lightly trypsinised using the DetachKit (PromoCell, UK) according to the manufacturer’s instructions.

### Pellet culture for chondrogenic differentiation

250,000 hMSCs were suspended in 500 μl of either growth or chondrogenic medium (Mesenchymal Stem Cell Chondrogenic Medium; PromoCell, UK) and centrifuged at 450 x g for 10 min in a 15 ml polypropylene conical tube. Cells were maintained at 37°C and 5% CO_2_ in the conical tubes with loosened caps for gas exchange. Medium was changed every 2-3 days. 

### Micromass for chondrogenic differentiation

250,000 hMSCs were suspended in 200 μl of growth medium in a 96-well round bottom suspension culture plate (Nunc, UK). After 48 h, spheroids began to form spontaneously and cells were fed with either growth or chondrogenic medium. Medium was changed every 2-3 days.

### Type II collagen hydrogels for chondrogenic differentiation

Bovine articular cartilage-derived type II collagen (BD Biosciences, UK) was diluted on ice to a final concentration of 1.5 mg/ml in either growth or chondrogenic medium and 2.5% (v/v) HEPES Buffer. The solution was neutralised (pH 7.4) with NaOH and 250,000 hMSCs were seeded in a 200 μl total volume in a 96-well plate. The hydrogels were incubated for 4-5 h at 37°C and 5% CO_2_ and then an additional 125 μl of medium was added to each well. Medium was changed every 2-3 days.

### Histology

In order to evaluate cell morphology and the establishment of an extracellular matrix rich in sulphated glycosaminoglycans (sGAGs) and collagen, histological staining was performed. After 21 days in culture, the samples were washed in PBS, fixed in 3.7% (w/v) paraformaldehyde, washed thoroughly, and stored at 4°C in PBS until embedding. Paraffin embedded samples were sectioned to 10 μm thickness, dewaxed, and stained with haematoxylin/eosin, Alcian blue, and picrosirius red according to standard methods.

### RNA extraction and cDNA synthesis

Cells were washed in PBS and 350 μl RLT supplemented with 1% (v/v) β-mercaptoethanol was added. The samples was vigorously lysed with pipetting and vortexing and stored at -20°C prior to RNA extraction using an RNeasy mini kit (Qiagen, UK) according to the manufacturer’s instructions. RNA quality and quantity were assessed on a NanoDrop 2000c. First-strand cDNA was synthesised using Superscript III First-Strand Synthesis kit (Invitrogen, UK) according to the manufacturer’s instructions. 100 ng total RNA was reverse-transcribed in a 20 μl reaction volume followed by an RNase H treatment. cDNA was diluted in dH_2_O prior to qPCR.

### Quantitative real-time polymerase chain reaction (qPCR)

Quantification of mRNA transcripts from three independent experiments (in technical triplicate) occurred after 1, 2, 4, 7, 14, and 21 days. A day 0 sample, hMSCs prior to trypsinisation, was used to normalise integrin subunit expression. Quantitative PCR was performed in a 10 μl reaction on a Rotorgene 6000 (Qiagen, UK) using a Platinum SYBR Green Supermix-UDG kit (Invitrogen, UK) according to the manufacturer’s instructions. Primer sequences (Table S1) to the phenotype markers and integrin subunits were obtained from PrimerBank (Harvard, USA; http://pga.mgh.harvard.edu/primerbank/), with the exception of primers to *ACAN*, *COL6A1*, *ITGA4*, and *ITGA6*, which were purchased from Qiagen (Quantitect). Thermocycling was as follows: 50°C for 2 min, 95°C for 2 min, 40 cycles of 95°C for 15 s and 62°C for 30 s, and a high resolution melt curve from 70-95°C. For the Quantitect primers, the annealing temperature was 63°C instead of 62°C. All primers were validated with a standard curve to ensure an efficiency of 0.9-1.0 and amplicons were run on an agarose gel to ensure a single product of the correct size. *GAPDH* was used as a house-keeping gene (*p* > 0.7 over the time-course, no significant differences in GAPDH detected by Western blotting). Data for the integrin subunits were plotted as 2^-ΔΔCt^ (i.e.- relative to *GAPDH* and to hMSCs prior to trypsinisation), except when this value was negative, where they were plotted as -1/2^-ΔΔCt^. Data for the phenotype markers were plotted as 2^-ΔCt^ (i.e.- relative to *GAPDH*) because the genes were typically not detected in hMSCs.

### shRNA silencing of *ITGB8*

Lentiviral particles containing shRNA to silence *ITGB8* and control (scrambled) shRNA were purchased from Santa Cruz (Germany). Transduction conditions were optimised using Cop-GFP lentiviral particles. hMSCs were seeded in 12-well plates at 6,000 cells/cm^2^ and grown to 50% confluency. They were transduced with 10 μl/ml virus in 5 μg/ml polybrene in growth medium. After 24 h, the medium was changed and the cells were allowed to recover for a further 24 h. They were then switched to selection medium, containing 4 μg/ml puromycin. After three days, cells were pooled, assayed for transduction efficiency, and cultured under standard growth conditions until differentiation experiments. Differentiation was performed in the micromass model and transcript quantification occurred after 0, 4, 7, and 21 days.

### Western blotting of integrin β8

hMSCs were washed in PBS and lysed in RIPA buffer (Sigma, UK) supplemented with protease (Roche, UK) and phosphatase (Sigma, UK) inhibitors. The lysate was clarified by centrifugation at 4°C and total protein was quantified with a Bradford assay (Sigma, UK). 15 μg total protein in Laemmli’s Sample Buffer was loaded on a 10% SDS-PAGE gel. Protein was transferred to a PVDF membrane under wet transfer conditions. The membrane was blocked with 5% (w/v) BSA in TBS/T and incubated in antibodies against integrin β8 (1/200; sc-6638, Santa Cruz, Germany) and GAPDH (1/5000; Clone 2D4A7, Thermo Fisher, UK) overnight at 4°C. After washing, the membrane was incubated in secondary antibodies against IgG (anti-goat 1/5000 and anti-mouse 1/5000; Li-Cor Biosciences, UK) labelled with IRDye. The blot was visualised on an Odyssey CLx with Image Studio software (Li-Cor Biosciences, UK).

### Statistical analysis

Student’s t-test and ANOVA were used to determine statistical significance (*p* < 0.05). Quantitative PCR data are from three independent experiments performed in technical triplicates. Relative expression was calculated using the ΔΔC_t_ method (relative to *GAPDH* and day 0 hMSCs), and down-regulation is presented as the negative inverse.

## Results

### Cartilage phenotype markers during chondrogenic differentiation

**Figure 1:**
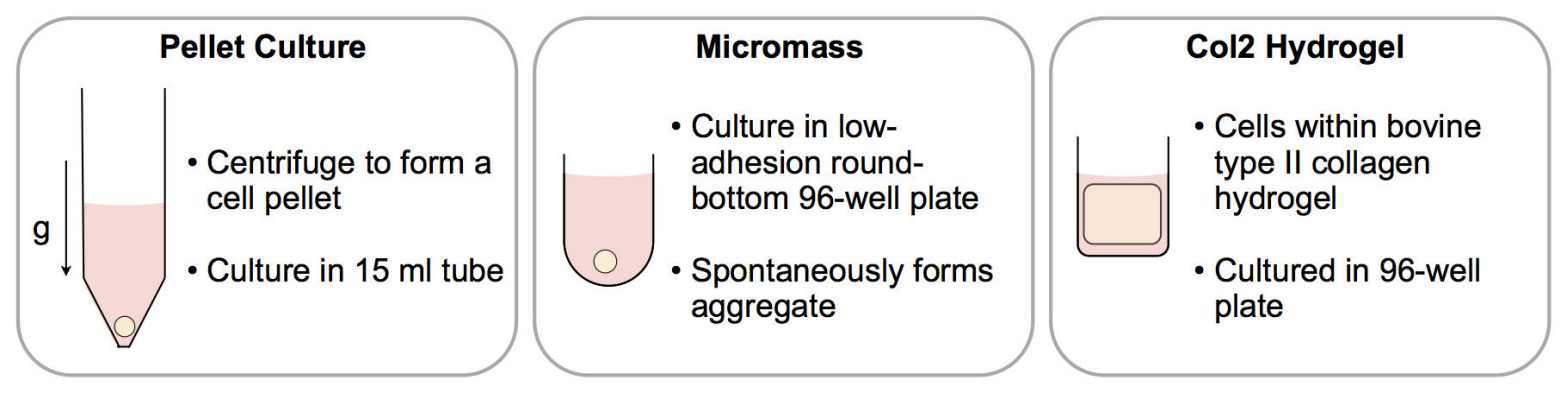
To study the changing expression of integrins during chondrogenic differentiation, human mesenchymal stem cells (hMSCs) were cultured in three different chondrogenesis models: pellet culture, micromass, and type II collagen hydrogels under two different conditions: growth medium and chondrogenic medium.

**Figure 2:**
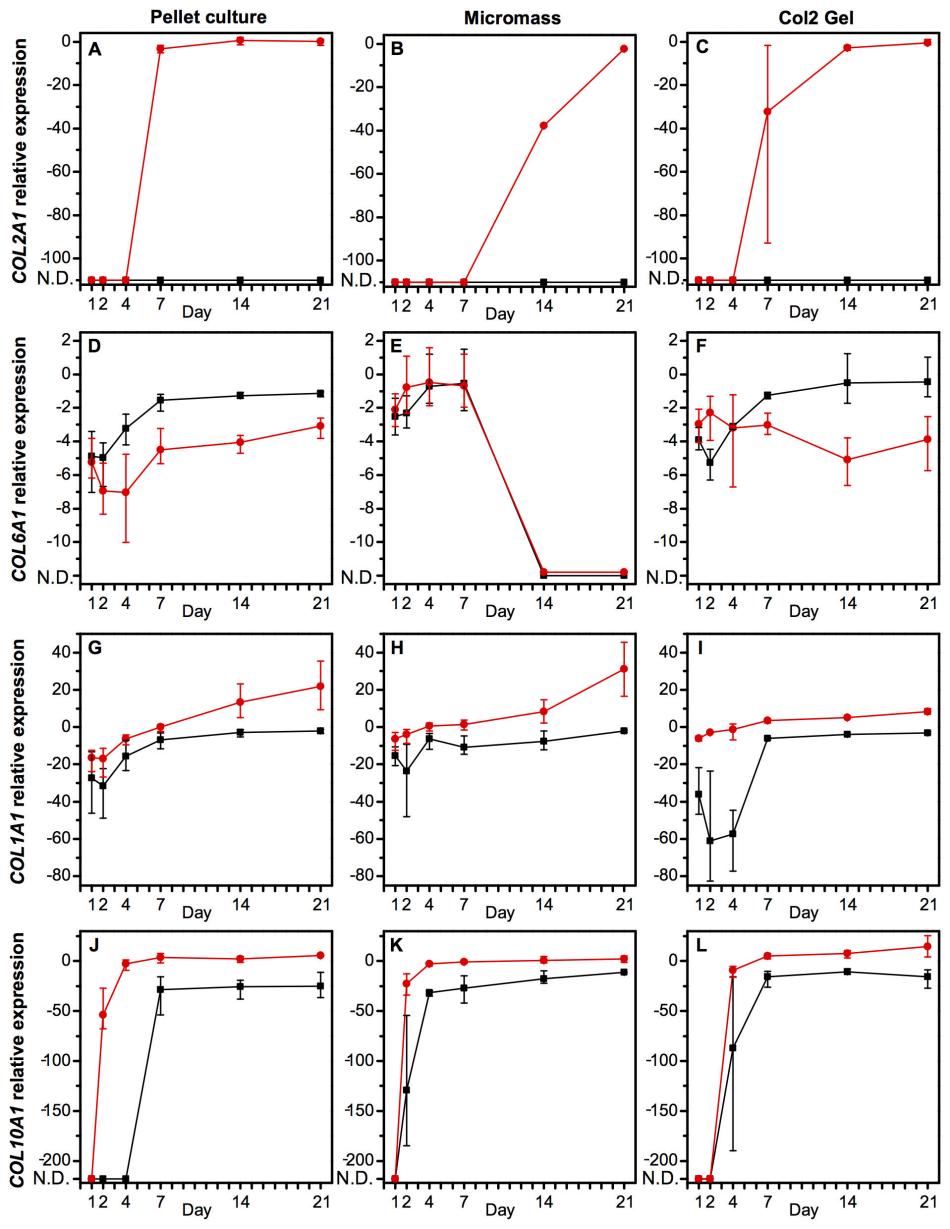
Quantitative PCR established mRNA expression in hMSCs in three different chondrogenesis models (pellet culture, micromass culture, or a type II collagen hydrogel) in either growth or chondrogenic medium over a time-course of 21 days. A high expression of COL2A1 (A-C) and COL6A1 (D-F) are markers of a hyaline cartilage-like extracellular and pericellular matrix, respectively. COL1A1 (G-I) and COL10A1 (J-L) are markers of non-hyaline cartilage. Data are transcript expression relative to GAPDH as an internal control (N.D.: not detected). Black squares: hMSCs cultured in growth medium, red circles: hMSCs cultured in chondrogenic medium. Data are the mean values of *N*=3 independent experiments performed in technical triplicate. Error bars indicate the range of values. Statistical significance is shown in Figures S15-S16.

**Figure 3:**
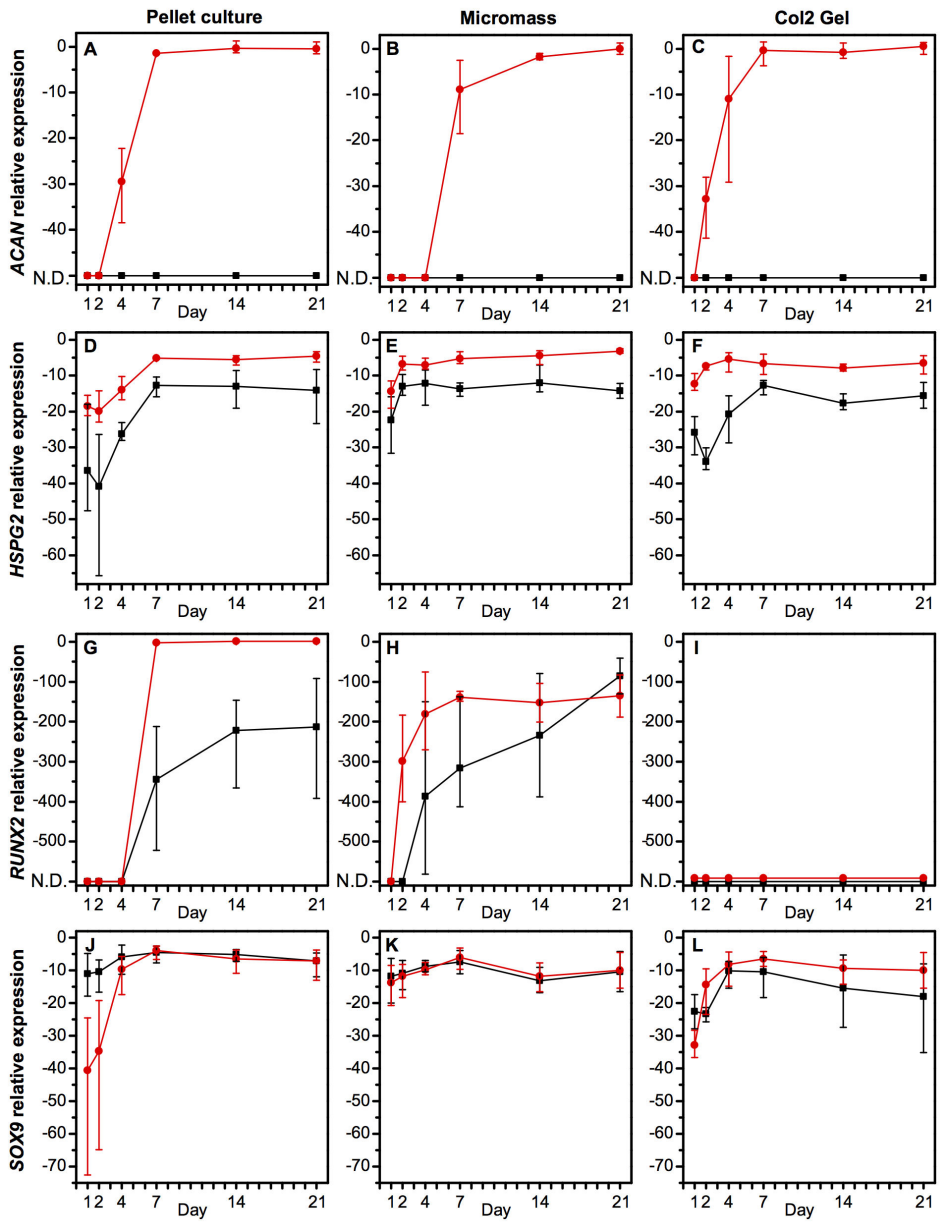
Quantitative PCR established mRNA expression in hMSCs in three different chondrogenesis models (pellet culture, micromass culture, or a type II collagen hydrogel) in either growth or chondrogenic medium over a time-course of 21 days. A high expression of ACAN (A-C) and *HSPG2* (D-F) are markers of a hyaline cartilage ECM and pericellular matrix, respectively. RUNX2 (G-I) and SOX9 (J-L) are markers of osteogenesis and chondrogenesis, respectively. Data are transcript expression relative to GAPDH as an internal control (N.D.: not detected). Black squares: hMSCs cultured in growth medium, red circles: hMSCs cultured in chondrogenic medium. Data are the mean values of *N*=3 independent experiments performed in technical triplicate. Error bars indicate the range of values. Statistical significance is shown in Figures S15-S16.

Before determining the changing integrin expression during chondrogenesis, we sought an appropriate *in vitro* model that resulted in a high expression of chondrogenic markers and the establishment of a hyaline cartilage-like extracellular matrix (ECM). We therefore used qPCR to characterise three different chondrogenesis models (Figure 1; pellet culture, micromass culture, and a type II collagen hydrogel) for the expression of *COL1A1* (type I collagen), *COL2A1* (type II collagen), *COL6A1* (type VI collagen), *COL10A1* (type X collagen), *ACAN* (aggrecan), *HSPG2* (perlecan), *RUNX2*, and *SOX9* (Figures 2-3), and also used histology to view an ECM rich in collagen and sulphated glycosaminoglycans (Figure S1). In all experiments, results are reported as statistically significant when *p* < 0.05.

Figure 2 demonstrates the temporally changing expression of four types of collagen mRNA in hMSCs cultured in either growth or chondrogenic medium in the three different chondrogenesis models. Only hMSCs cultured in chondrogenic medium expressed *COL2A1* (Figure 2A-C). Cells cultured in a micromass had delayed expression of this key marker of articular hyaline cartilage ECM, but a similar level of the transcript was achieved in all models after 21 days. *COL6A1*, the defining component of the pericellular matrix, was relatively unchanged in hMSCs in a pellet culture or a type II collagen hydrogel (Figure 2D-F). However, in the micromass, it was down-regulated to non-detectable levels after seven days. For *COL1A1*, there were no significant differences between the chondrogenesis models, but as to be expected, chondrogenic medium resulted in an up-regulation over time (Figure 2G-I). *COL10A1* was highly up-regulated over time and to a significantly higher degree in cells in chondrogenic medium (Figure 2J-L), while for this gene, there were no significant differences between the chondrogenesis models.

Figure 3 demonstrates the temporal changes of mRNA expression of four other phenotype markers in hMSCs cultured in either growth or chondrogenic medium in the three different chondrogenesis models. For *ACAN*, which was only expressed in the presence of chondrogenic medium, there were no significant differences between the chondrogenesis models, except a slight delay of transcript expression in the micromass (Figure 3A-C). *HSPG2*, the gene encoding perlecan, a component of the pericellular matrix, was unaffected by the chondrogenesis system, but was expressed at significantly higher levels in cells cultured in chondrogenic medium (Figure 3D-F). *RUNX2*, a transcription factor central in osteogenesis, but with an additional role in chondrogenesis, was highly up-regulated over time in the pellet culture and micromass but was undetectable in the type II collagen hydrogels (Figure 3G-I). *SOX9*, a transcription factor central but not exclusive to chondrogenesis, was unaffected by either the chondrogenesis model or the medium conditions over time (Figure 3J-L).

### hMSCs and chondrocytes express different integrin subunits

**Figure 4:**
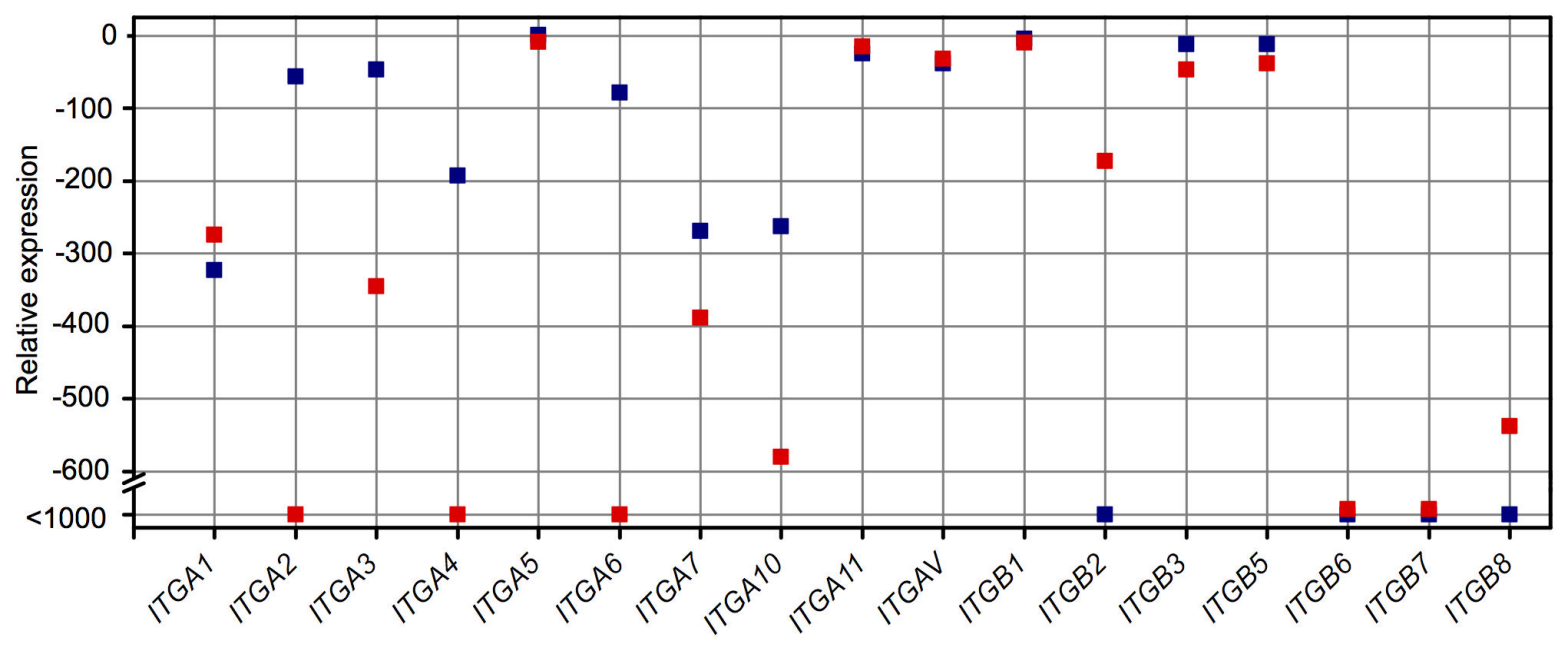
Quantitative PCR established mRNA expression of integrin subunits in hMSCs in standard culture conditions (48 h after seeding) and chondrocytes derived from cartilage. Most subunits were down-regulated in chondrocytes compared to hMSCs, with the exception of *ITGA1*, *ITGB2*, and ITGB8, which were significantly up-regulated, and ITGA5, *ITGA11*, ITGAV, and ITGB1, which were unchanged. Blue: hMSCs, red: chondrocytes. Data are the mean expression, relative to GAPDH of *N*=2 independent experiments performed in technical triplicate.

In order to determine the differences between integrin subunits expressed in hMSCs and chondrocytes, RNA was isolated from hMSCs in standard culture conditions (48 h after seeding) and from normal human articular chondrocytes (PromoCell HCH Pellet, UK). Figure 4 shows the relative expression of ten α subunits and seven β subunits. *ITGA2*, *ITGA3*, *ITGA4*, *ITGA6*, *ITGA7*, *ITGA10*, *ITGB3*, and *ITGB5* mRNA levels were statistically significantly higher in hMSCs than in chondrocytes. *ITGA1*, *ITGB2*, and *ITGB8* mRNA levels were statistically significantly higher in chondrocytes than in hMSCs. Expression of *ITGA5*, *ITGA11*, *ITGAV*, and *ITGB1* were unchanged between the two cell types. *ITGB6* and *ITGB7* were not detected in either hMSCs or chondrocytes.

### Integrin expression in chondrogenesis

**Figure 5:**
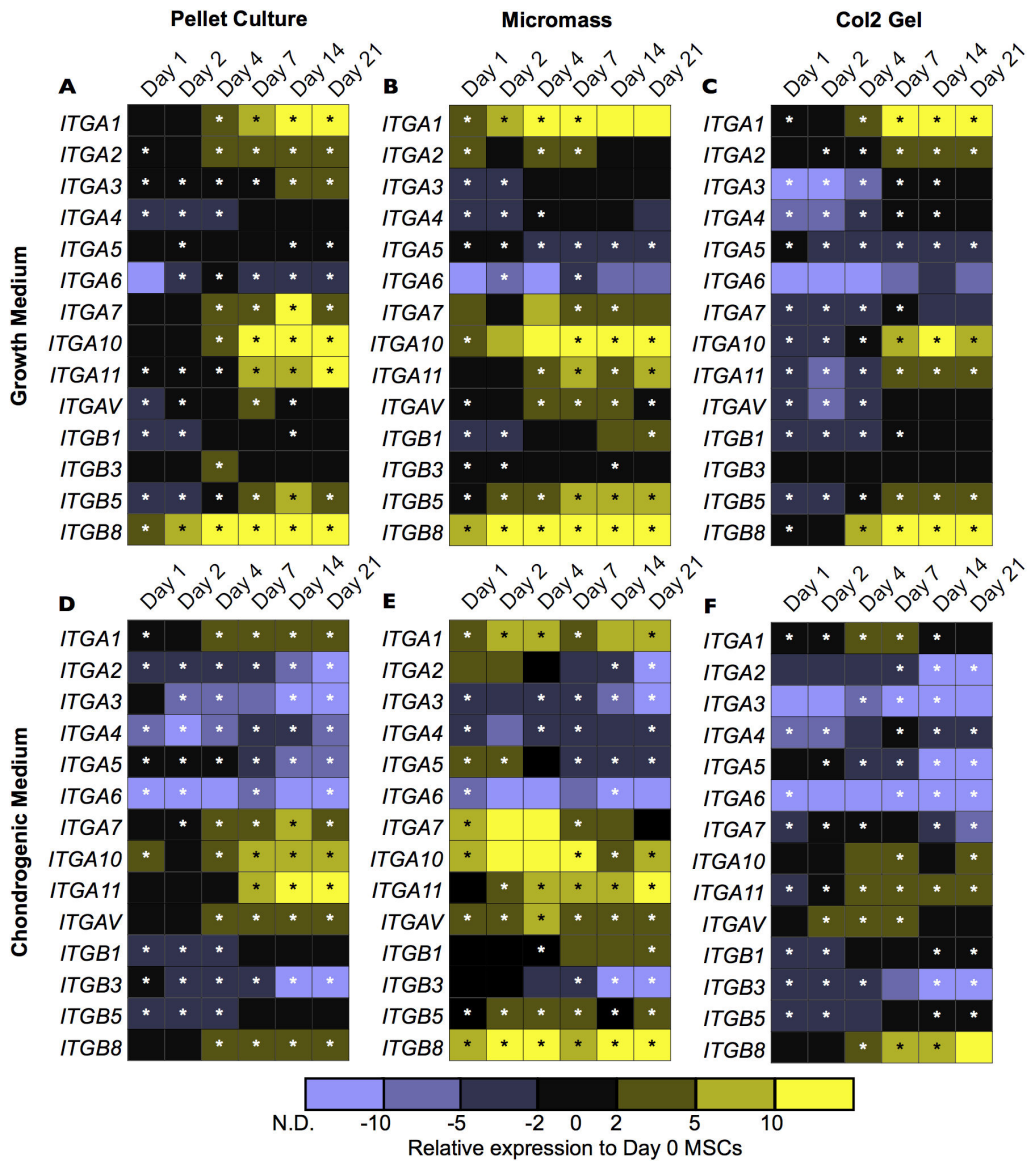
Quantitative PCR established mRNA expression of integrin subunits in hMSCs cultured in three different chondrogenesis models (pellet culture, micromass culture, or a type II collagen hydrogel) in either growth or chondrogenic medium over a time-course of 21 days. In general, integrin mRNA was down-regulated in chondrogenic medium and there were similar expression patterns between the different chondrogenesis conditions. Each square represents mean expression relative to GAPDH and to day 0 hMSCs on tissue culture plastic in growth medium of *N*=3 independent experiments performed in technical triplicate. Down-regulation is represented in purple (N.D.: not detected) and up-regulation is represented in yellow. Statistically significant changes (*p* < 0.05) compared to day 0 are denoted by an asterisk (*). Line charts depicting the means and range of values are in the Figures S2-S8, and statistical significance is in Figures S12-S14.

To better understand how the expression of integrin subunits changes during chondrogenic differentiation of hMSCs, a time-course of transcript levels over 21 days was established with qPCR. Figure 5 shows the expression of 14 integrin subunits relative to *GAPDH* as an internal control and to day 0 hMSCs (prior to trypsinisation). 

Three conditions and their effect on integrin expression were studied concurrently: the effect of the culture medium, the effect of the chondrogenesis model, and the effect of the time-course. In general, cells in growth medium (Figure 5A-C) had higher integrin expression than in chondrogenic medium (Figure 5D-F). The medium composition had a significant effect (*p* < 0.05) on all subunits measured except for *ITGA6*, *ITGA7*, and *ITGA11* in the pellet culture, *ITGA4*, *ITGA6*, *ITGA7*, *ITGA10*, and *ITGB1* in the micromass culture, and *ITGA7* and *ITGB1* in the type II collagen hydrogel. In comparing the three chondrogenesis models, integrin subunit expression was also well-conserved. For cells cultured in growth medium, only *ITGA7* expression was different (*p* < 0.05) during the time-course between the different chondrogenesis models. For cells cultured in chondrogenic medium, only *ITGA5, ITGA6*, and *ITGB3* expression were different between the chondrogenesis models. The time-course had the greatest effect on the expression of integrin subunit mRNA. In growth medium, statistically significant up-regulation was observed for *ITGA1* (pellet culture and type II collagen hydrogel), *ITGA3* (pellet culture and type II collagen hydrogel), *ITGA4* (type II collagen hydrogel), *ITGA7* (pellet culture), *ITGA10* (pellet culture and type II collagen hydrogel), *ITGA11* (all chondrogenesis models), *ITGAV* (all chondrogenesis models), *ITGB1* (all chondrogenesis models), *ITGB5* (all chondrogenesis models), and *ITGB8* (all chondrogenesis models). Down-regulation was not statistically significant for any integrin subunits in hMSCs cultured growth medium. In chondrogenic medium, there was generally less up-regulation of the integrin subunits than in growth medium. Statistically significant up-regulation was observed for *ITGA4* (pellet culture), *ITGA7* (pellet culture), *ITGA11* (all chondrogenesis models), *ITGAV* (pellet culture), *ITGB5* (pellet culture), and *ITGB8* (all chondrogenesis models). Statistically significant down-regulation was observed for *ITGA2* (micromass), *ITGA3* (pellet culture), *ITGA5* (all chondrogenesis models), *ITGA6* (type II collagen hydrogel), and *ITGB3* (all chondrogenesis models).

### Knockdown of *ITGB8* interferes with chondrogenic phenotype

**Figure 6:**
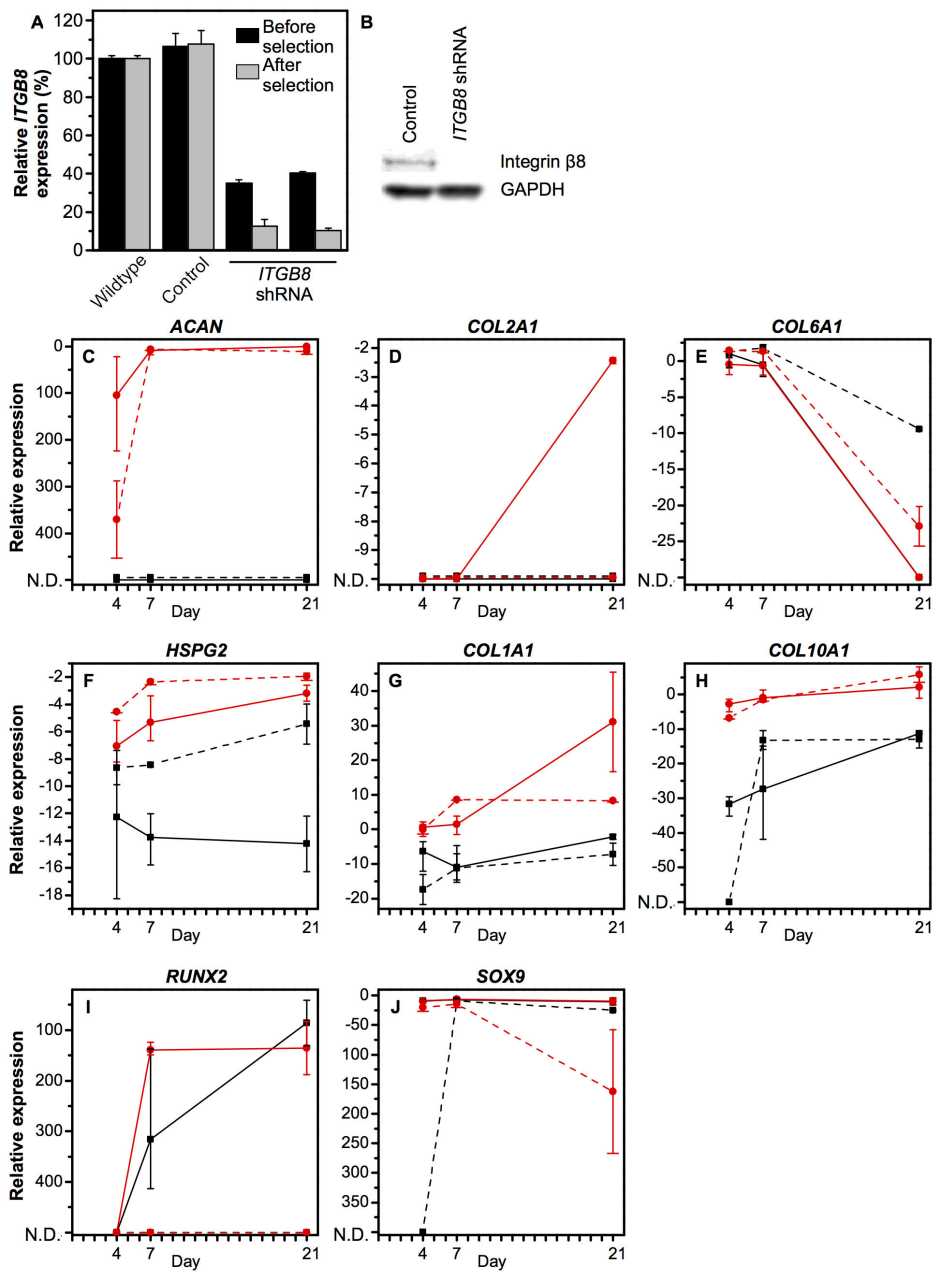
Lentiviral particles containing shRNA against ITGB8 were used to investigate its role during *in* *vitro* chondrogenesis in the micromass model. A knockdown efficiency of 89% after puromycin selection was determined by qPCR (A) and confirmed by Western blot (B). All chondrogenic phenotype markers were affected by reduced ITGB8. In particular, hyaline cartilage ECM markers ACAN (C) and COL2A1 (D) were down-regulated, while pericellular matrix markers COL6A1 (E) and *HSPG2* (F) were up-regulated. Non-hyaline cartilage markers COL1A1 (G) and COL10A1 (H) were down-regulated. The transcription factors RUNX2 (I) and SOX9 (J) were also down-regulated. Black squares: growth medium, red circles: chondrogenic medium. Solid lines: wildtype, dashed line: ITGB8 knockdown. Data represent mean and range of values relative to GAPDH for *N*=2 or 3 experiments performed in technical triplicate. Statistical significance is in Figure S18.

An shRNA lentiviral construct was used to create a stable knockdown of *ITGB8*, an integrin subunit known to be involved in the release of insoluble TGF-β from its latency associated peptide and therefore thought to be important in chondrogenic differentiation [21]. Our first experiments had demonstrated that it was one of the few integrin subunits consistently up-regulated in chondrogenic differentiation regardless of the medium composition or the chondrogenesis model. Figure 6A depicts the knockdown efficiency before and after puromycin selection. After selection, quantitative PCR showed a silencing efficiency of 89% and the protein was undetectable by Western blotting (Figure 6B). Following expansion, the cells were differentiated in the micromass model of chondrogenesis. 

Integrin subunit expression during chondrogenic differentiation was generally unaffected in the integrin β8 knockdown compared to the wildtype (Figures S9-S11). After 21 days, there were no statistically significant differences in the α subunits. The β subunits were more affected, with *ITGB3* expression undetectable after four days. Interestingly, many integrin subunits were up-regulated in the integrin β8 knockdown hMSCs at day 0 (prior to formation of the micromass). This effect was statistically significant for *ITGA3*, *ITGA4*, *ITGA6*, *ITGA7*, *ITGA11*, *ITGAV*, *ITGB1*, and *ITGB5* (all up-regulated in the *ITGB8* knockdown), and *ITGA5* (down-regulated in the *ITGB8* knockdown).

All phenotype markers were affected by knocking down integrin β8 (Figure 6C-J). *ACAN* was still only expressed in chondrogenic medium, but it was significantly down-regulated in the knockdown after 4 days compared to wildtype hMSCs. *COL2A1* expression had a striking change in the integrin β8 knockdown, where it was not detected at any point. The two pericellular matrix markers, *COL6A1* and *HSPG2*, were significantly up-regulated compared to wildtype in both growth and chondrogenic medium, with a greater up-regulation observed in growth medium. *COL1A1* was significantly down-regulated in chondrogenic medium after 21 days but was unaffected in growth medium. *COL10A1* was down-regulated compared to the wildtype in growth medium at day 7, but was expressed similarly by day 21. In chondrogenic medium, it was unaffected by the knockdown of integrin β8. *RUNX2* expression was abolished by knocking down integrin β8 in both growth and chondrogenic medium at all time points. *SOX9* was significantly down-regulated after 21 days in chondrogenic medium but was unaffected in growth medium. 

## Discussion

Tissue engineering strategies offer a promising treatment for diseased or damaged cartilage. However, they have so far failed to deliver on this promise, and scaffolds are frequently marred by their inability to support initial hMSC attachment and subsequent differentiation into chondrocytes. Our aim was to develop a better understanding of the changing integrin expression during chondrogenic differentiation. This could lead to an improved scaffold design, incorporating the ligands required to promote initial attachment and drive chondrogenic signalling to ultimately create a therapeutically useful cartilage construct. We used qPCR and the *in vitro* chondrogenic differentiation models commonly used in cartilage tissue engineering to ensure our results were translatable to other studies. We showed how integrin transcript expression changes during chondrogenic differentiation of hMSCs in three chondrogenesis models and found a new role for integrin β8 in the establishment of a chondrogenic phenotype.

We began by examining the expression of key phenotype markers in hMSCs undergoing chondrogenic differentiation in three relevant *in vitro* models. We found that the phenotype markers were remarkably well-conserved in the three models, with only *COL6A1* and *RUNX2* differentially expressed. The disruption of *RUNX2*, as seen in the type II collagen hydrogel, has been shown to block chondrocyte hypertrophy and the progression of osteoarthritis [22]. Two commonly used indicators of articular cartilage phenotype, the *COL2A1*/*COL1A1* and *COL2A1*/*COL10A1* ratios, were statistically similar (*p* > 0.05) in the different chondrogenesis models at all time points. We then quantified the changing integrin expression of hMSCs undergoing chondrogenesis. While the 21 day time-course was not long enough to produce mature cartilage, the trends of the integrin transcript expression we measured in differentiating hMSCs were in agreement with the expression we found in cells derived from healthy human articular cartilage. Generally, we found that integrin expression was higher in cells cultured in growth medium than in chondrogenic medium and, like the phenotype markers, was widely conserved across the chondrogenesis models. The most striking changes were in the changing expression over the 21 day time-course. The hMSCs had significantly changing transcript expression for most integrins, which should come as no surprise, given the role integrins play in virtually every aspect of development.

While this is one of the most extensive studies quantifying changing integrin expression in multiple chondrogenesis models over a 21 day time-course, it is by no means the first example of integrins and integrin ligands affecting MSCs and chondrogenesis. The expression of a number of cell surface receptors has been previously reported in MSCs [23]. Some studies have looked at changing integrin expression in chondrogenesis and in dedifferentiation of chondrocytes [24,25]. For example, the β1 and α3 subunits have been implicated in dedifferentiation, followed by an increase of fibronectin in the ECM [26]. Cartilage sections have also been stained for integrins. It has been reported that the most abundant integrin in chondrocytes is α5β1 [18], and that only αv is differentially expressed in the different cartilage zones [27,28]. Our results are in line with many of the studies examining specific integrin expression. For example, integrin α1 was previously detected in human articular cartilage, while integrin α3 was not [27]. We observed up-regulation of *ITGA1* and down-regulation of *ITGA3* in chondrogenesis, in agreement with this result.

*ITGB8* was one of the only integrin subunits consistently up-regulated in all chondrogenesis models in this study. Given its known role in the mechanical and proteolytic release of TGF-β from its latency complex [21], we sought to demonstrate its role in chondrogenesis. An 89% mRNA knockdown led to non-detectable expression of *COL2A1*, the defining marker of hyaline cartilage, demonstrating the importance of this integrin in the chondrogenic differentiation of hMSCs. It also resulted in an up-regulation of the pericellular matrix markers *COL6A1* and *HSPG2*, and in both cases, this increase was more pronounced in growth medium than in chondrogenic medium. How this integrin β8 knockdown is affecting TGF-β signalling remains to be seen, but it may have to do with the source of TGF-β. There is some evidence that while TGF-β3 has a positive effect, TGF-β1 signalling may suppress chondrogenic differentiation [29]. The integrin αvβ8 knockdown would have diminished the ability of the cell to activate insoluble (cell-secreted) TGF-β but would not have necessarily interfered with its binding of the soluble (medium supplemented, TGF-β3) form of the growth factor. This could at least partially explain why the effects of the knockdown of integrin β8 on cell phenotype were affected by the medium composition. Future work could include characterising the effect of the TGF-β isoforms on integrin αvβ8-mediated signalling in chondrogenic differentiation. 

A number of studies have demonstrated that incorporating integrin ligands in a scaffold can influence cell behaviour. For example, IKVAV, a laminin-derived integrin ligand, has been shown to support hMSC viability [30]. In our experiments, we found that *ITGA3* and *ITGA6*, which are known to heterodimerise with *ITGB1* (along with *ITGB4* in the case of *ITGA6*) to bind laminin, were typically down-regulated in chondrogenic differentiation. This agrees with the finding that hMSCs can be supported by IKVAV but suggests that chondrocytes will not. As a second example, when GFOGER, a collagen mimetic peptide capable of binding cells *via* β1 integrins was incorporated in poly(ethylene glycol) (PEG) hydrogels, hMSCs underwent chondrogenic differentiation to a greater extent than in the hydrogels alone [31]. This is also in agreement with our results, which found relatively constant expression of the collagen-binding integrins, suggesting both hMSCs and chondrocytes would respond well to extracellular collagen. Finally, fibronectin and its peptide derivatives are frequently used to improve stable cell adhesion though some studies demonstrate a negative effect on chondrogenesis when RGD is incorporated in scaffolds [32,33]. In this study, we found some of the fibronectin-binding integrins (in particular, *ITGAV*) maintained relatively constant expression during chondrogenesis. The presence of these receptors indicates that both hMSCs and chondrocytes might maintain their phenotype in the presence of fibronectin. This is reasonable, considering fibronectin is present in the ECM throughout differentiation and in mature chondrocytes [34]. It would be of particular interest to cater to the temporally changing integrin expression to guide stem cells to become chondrocytes. Integrin ligands, catering to the temporally changing integrin expression determined in this study, could be incorporated in tissue engineering scaffolds to influence cell behaviour. This concept has been attempted in adipogenesis and osteogenesis, but experiments targeting specific integrins in chondrogenesis have thus far primarily focused on preventing dedifferentiation of mature chondrocytes [35].

## Conclusion

In conclusion, we have completed a characterisation of integrin expression in three *in vitro* chondrogenesis models. 

We found that integrin expression generally decreased in chondrogenic differentiation and that the expression of most subunits was temporally regulated. We further examined the role for integrin β8 and found that its knockdown resulted in an up-regulation of pericellular matrix synthesis and an elimination of *COL2A1* expression. Having studied integrin expression in multiple chondrogenesis models and demonstrated a role for integrin β8 in chondrogenesis, this study has improved our understanding of integrin expression in chondrogenic differentiation and the role of the ECM in influencing cell behaviour. It can inform the future design of tissue engineering scaffolds to include ligands to cater to the changing adhesion requirements of hMSCs in chondrogenic differentiation.

## Supporting Information

Figure S1 **Histological sections of hMSCs cultured in three chondrogenesis models (pellet culture, micromass, and type II collagen hydrogel) for 21 days in either growth or chondrogenic medium.** Haematoxylin and eosin (H&E) staining demonstrates cell morphology, picrosirius red (SR) stains collagen, and Alcian blue (AB) stains sulphated glycosaminoglycans. Scale: 50 μm.
(TIFF)

Figure S2 **Quantitative PCR established mRNA expression of integrin subunits *ITGA1* (**A**-**C**) and *ITGA2* (**D**-**F**) in hMSCs cultured in three different chondrogenesis models (pellet culture, micromass culture, or a type II collagen hydrogel) in either growth (black squares) or chondrogenic (red circles) medium over a time-course of 21 days.** Each point represents mean expression relative to *GAPDH* of *N*=3 independent experiments, and error bars represent the range of values. Statistical significance is in Figures S12-S14.
(TIFF)

Figure S3 **Quantitative PCR established mRNA expression of integrin subunits *ITGA3* (**A**-**C**) and *ITGA4* (**D**-**F**) in hMSCs cultured in three different chondrogenesis models (pellet culture, micromass culture, or a type II collagen hydrogel) in either growth (black squares) or chondrogenic (red circles) medium over a time-course of 21 days.** Each point represents mean expression relative to *GAPDH* of *N*=3 independent experiments, and error bars represent the range of values. Statistical significance is in Figures S12-S14.
(TIFF)

Figure S4 **Quantitative PCR established mRNA expression of integrin subunits *ITGA5* (**A**-**C**) and *ITGA6* (**D**-**F**) in hMSCs cultured in three different chondrogenesis models (pellet culture, micromass culture, or a type II collagen hydrogel) in either growth (black squares) or chondrogenic (red circles) medium over a time-course of 21 days.** Each point represents mean expression relative to *GAPDH* of *N*=3 independent experiments, and error bars represent the range of values. Statistical significance is in Figures S12-S14.
(TIFF)

Figure S5 **Quantitative PCR established mRNA expression of integrin subunits *ITGA7* (**A**-**C**) and *ITGA10* (**D**-**F**) in hMSCs cultured in three different chondrogenesis models (pellet culture, micromass culture, or a type II collagen hydrogel) in either growth (black squares) or chondrogenic (red circles) medium over a time-course of 21 days.** Each point represents mean expression relative to *GAPDH* of *N*=3 independent experiments, and error bars represent the range of values. Statistical significance is in Figures S12-S14.
(TIFF)

Figure S6 **Quantitative PCR established mRNA expression of integrin subunits *ITGA11* (**A**-**C**) and *ITGAV* (**D**-**F**) in hMSCs cultured in three different chondrogenesis models (pellet culture, micromass culture, or a type II collagen hydrogel) in either growth (black squares) or chondrogenic (red circles) medium over a time-course of 21 days.** Each point represents mean expression relative to *GAPDH* of *N*=3 independent experiments, and error bars represent the range of values. Statistical significance is in Figures S12-S14.
(TIFF)

Figure S7 **Quantitative PCR established mRNA expression of integrin subunits *ITGB1* (**A**-**C**) and *ITGB3* (**D**-**F**) in hMSCs cultured in three different chondrogenesis models (pellet culture, micromass culture, or a type II collagen hydrogel) in either growth (black squares) or chondrogenic (red circles) medium over a time-course of 21 days.** Each point represents mean expression relative to *GAPDH* of *N*=3 independent experiments, and error bars represent the range of values. Statistical significance is in Figures S12-S14.
(TIFF)

Figure S8 **Quantitative PCR established mRNA expression of integrin subunits *ITGB5* (**A**-**C**) and *ITGB8* (**D**-**F**) in hMSCs cultured in three different chondrogenesis models (pellet culture, micromass culture, or a type II collagen hydrogel) in either growth (black squares) or chondrogenic (red circles) medium over a time-course of 21 days.** Each point represents mean expression relative to *GAPDH* of *N*=3 independent experiments, and error bars represent the range of values. Statistical significance is in Figures S12-S14.
(TIFF)

Figure S9 **Quantitative PCR established mRNA expression of integrin subunits in hMSCs (wildtype: solid lines, *ITGB8* knockdown: dashed lines) cultured in a micromass in either growth (black squares) or chondrogenic (red circles) medium over a time-course of 21 days.** A) *ITGA1*, B) *ITGA2*, C) *ITGA3*, D) *ITGA4*, E) *ITGA5*, and F) *ITGA6* were unaffected by the *ITGB8* knockdown after 21 days. Each point represents mean expression relative to *GAPDH* and to hMSCs on tissue culture plastic in growth medium of *N*=2-3 independent experiments, and error bars represent the range of values. Statistical significance is in Figure S17.
(TIFF)

Figure S10 **Quantitative PCR established mRNA expression of integrin subunits in hMSCs (wildtype: solid lines, *ITGB8* knockdown: dashed lines) cultured in a micromass in either growth (black squares) or chondrogenic (red circles) medium over a time-course of 21 days.** A) *ITGA7*, B) *ITGA10*, C) *ITGA11*, and D) *ITGAV* were unaffected by the *ITGB8* knockdown after 21 days. Each point represents mean expression relative to *GAPDH* and to hMSCs on tissue culture plastic in growth medium of *N*=2-3 independent experiments, and error bars represent the range of values. Statistical significance is in Figure S17.
(TIFF)

Figure S11 **Quantitative PCR established mRNA expression of integrin subunits in hMSCs (wildtype: solid lines, *ITGB8* knockdown: dashed lines) cultured in a micromass in either growth (black squares) or chondrogenic (red circles) medium over a time-course of 21 days.** A) *ITGB1* was unaffected, B) *ITGB3* was undetectable, and C) *ITGB5* was unaffected by the *ITGB8* knockdown after 21 days. Each point represents mean expression relative to *GAPDH* and to hMSCs on tissue culture plastic in growth medium of *N*=2-3 independent experiments, and error bars represent the range of values. N.D.: not detected. Statistical significance is in Figure S17.
(TIFF)

Figure S12 **The statistical significance of the effect of the medium composition on integrin expression from Figure 5.** Student's t-test was used to determine *p*-values to indicate whether there was a statistically significant difference between cells cultured in growth or chondrogenic medium at each time-point in each of the three chondrogenesis models, A) pellet culture, B) micromass, or C) type II collagen hydrogel. *N*=3 independent experiments in technical triplicates.
(TIFF)

Figure S13 **The statistical significance of the effect of the chondrogenesis model on integrin expression from Figure 5.** A two-way ANOVA was used to determine *p*-values to indicate whether there was a statistically significant difference between cells cultured in pellet culture, micromass, or a type II collagen hydrogel in either A) growth medium, or B) chondrogenic medium. *N*=3 independent experiments in technical triplicates.
(TIFF)

Figure S14 **The statistical significance of the effect of the time-course on integrin expression from Figure 5.** Student's t-test was used to determine whether there was a statistically significant difference in integrin expression at each time-point in each of the three chondrogenesis models: A and D) pellet culture, B and E) micromass, or C and F) type II collagen hydrogel, when compared to hMSCs at day 0 in growth medium. *N*=3 independent experiments in technical triplicates.
(TIFF)

Figure S15 **The statistical significance of the effect of the medium composition on phenotype marker expression from Figures 2-3.** Student's t-test was used to determine *p*-values to indicate whether there was a statistically significant difference between cells cultured in growth or chondrogenic medium at each time-point in each of the three chondrogenesis models, A) pellet culture, B) micromass, or C) type II collagen hydrogel. *N*=3 independent experiments in technical triplicates.
(TIFF)

Figure S16 **The statistical significance of the effect of the chondrogenesis model on phenotype marker expression from Figures 2-3.** A two-way ANOVA was used to determine *p*-values to indicate whether there was a statistically significant difference between cells cultured in pellet culture, micromass, or a type II collagen hydrogel in either A) growth medium, or B) chondrogenic medium. *N*=3 independent experiments in technical triplicates.
(TIFF)

Figure S17 **The statistical significance of the effect of the knockdown of *ITGB8* on integrin expression.** Student's t-test was used to determine *p*-values to indicate whether there was a statistically significant difference in transcript expression between the knockdown and the wildtype in either A) growth medium, or B) chondrogenic medium. *N*=2-3 independent experiments in technical triplicates.
(TIFF)

Figure S18 **The statistical significance of the effect of the knockdown of *ITGB8* on phenotype marker expression from Figure 6.** Student's t-test was used to determine *p*-values to indicate whether there was a statistically significant difference in transcript expression between the knockdown and the wildtype in either A) growth medium, or B) chondrogenic medium. *N*=2-3 independent experiments in technical triplicates.
(TIFF)

Table S1 **Quantitative PCR primer sequences used in this study.**
(TIFF)
