# Supplementary figures and images for: The Changing Integrin Expression and a Role for Integrin β8 in the Chondrogenic Differentiation of Mesenchymal Stem Cells
Source: PLoS One. 2013 Nov 27;8(11):e82035. doi: 10.1371/journal.pone.0082035 (PMC3842320; doi:10.1371/journal.pone.0082035)

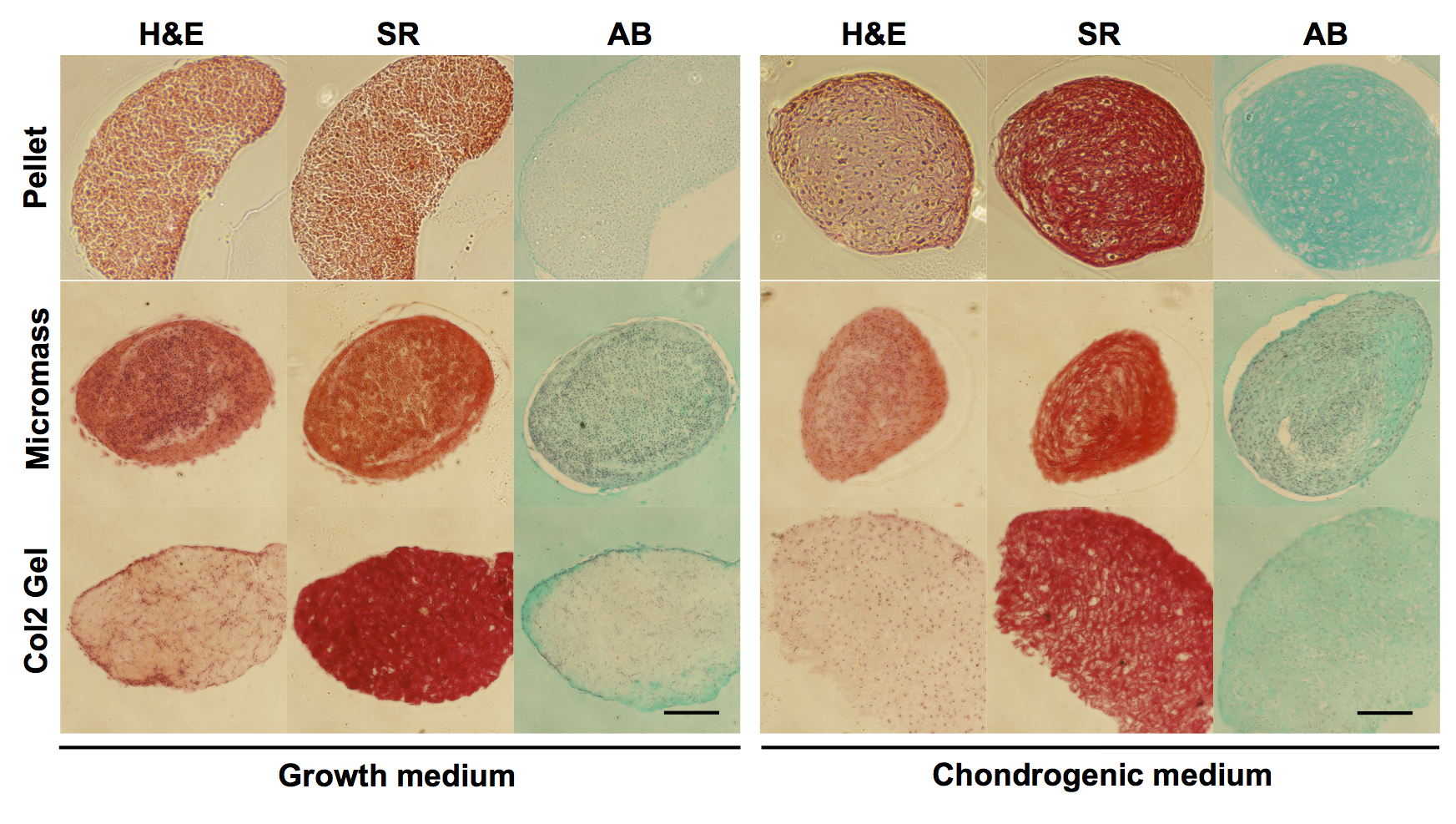

Supplement: Figure S1 — Histological sections of hMSCs cultured in three chondrogenesis models (pellet culture, micromass, and type II collagen hydrogel) for 21 days in either growth or chondrogenic medium. Haematoxylin and eosin (H&E) staining demonstrates cell morphology, picrosirius red (SR) stains collagen, and Alcian blue (AB) stains sulphated glycosaminoglycans. Scale: 50 μm. (TIFF) [file pone.0082035.s001.tiff]

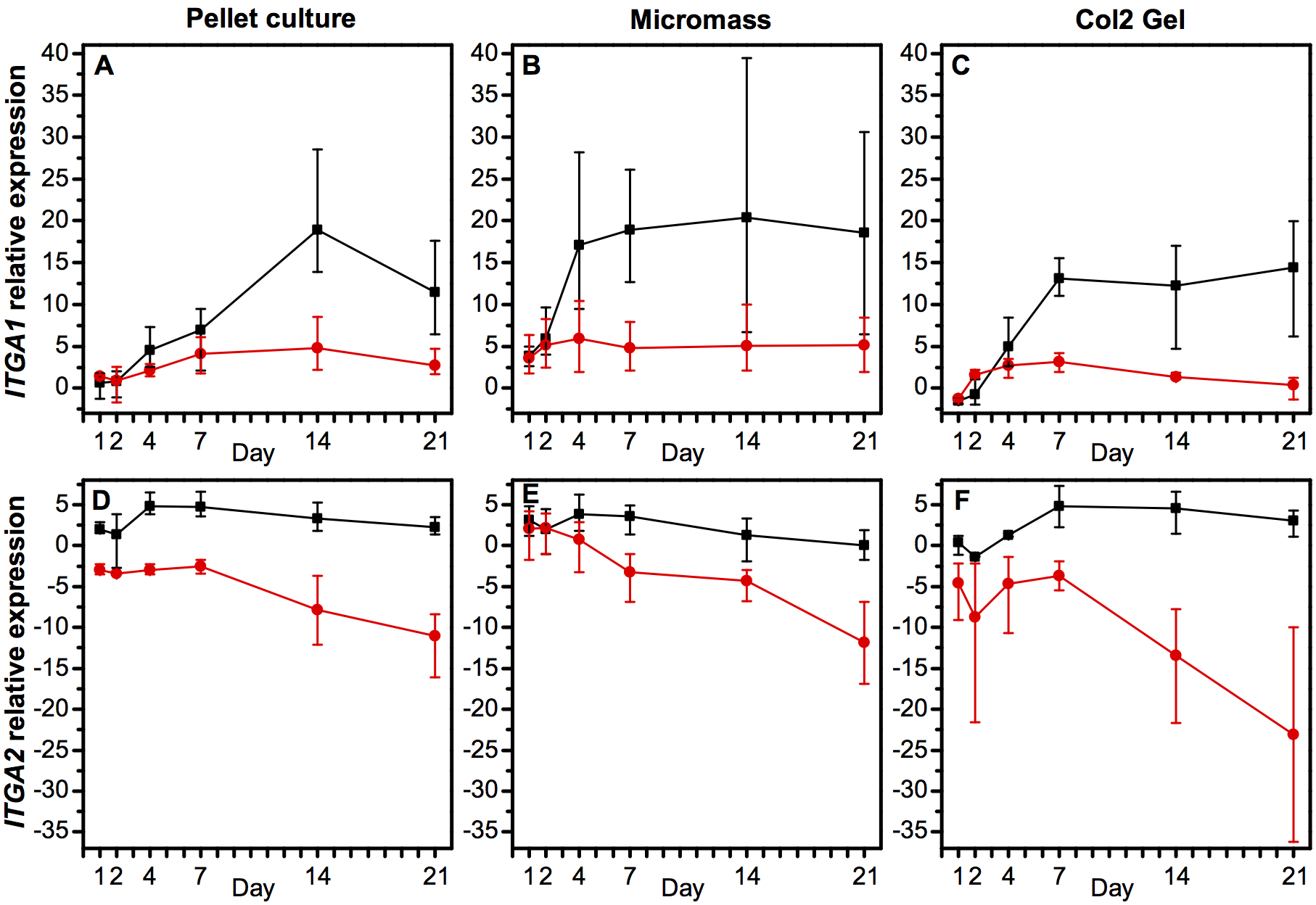

Supplement: Figure S2 — Quantitative PCR established mRNA expression of integrin subunits ITGA1 (A-C) and ITGA2 (D-F) in hMSCs cultured in three different chondrogenesis models (pellet culture, micromass culture, or a type II collagen hydrogel) in either growth (black squares) or chondrogenic (red circles) medium over a time-course of 21 days. Each point represents mean expression relative to GAPDH of N=3 independent experiments, and error bars represent the range of values. Statistical significance is in Figures S12-S14. (TIFF) [file pone.0082035.s002.tiff]

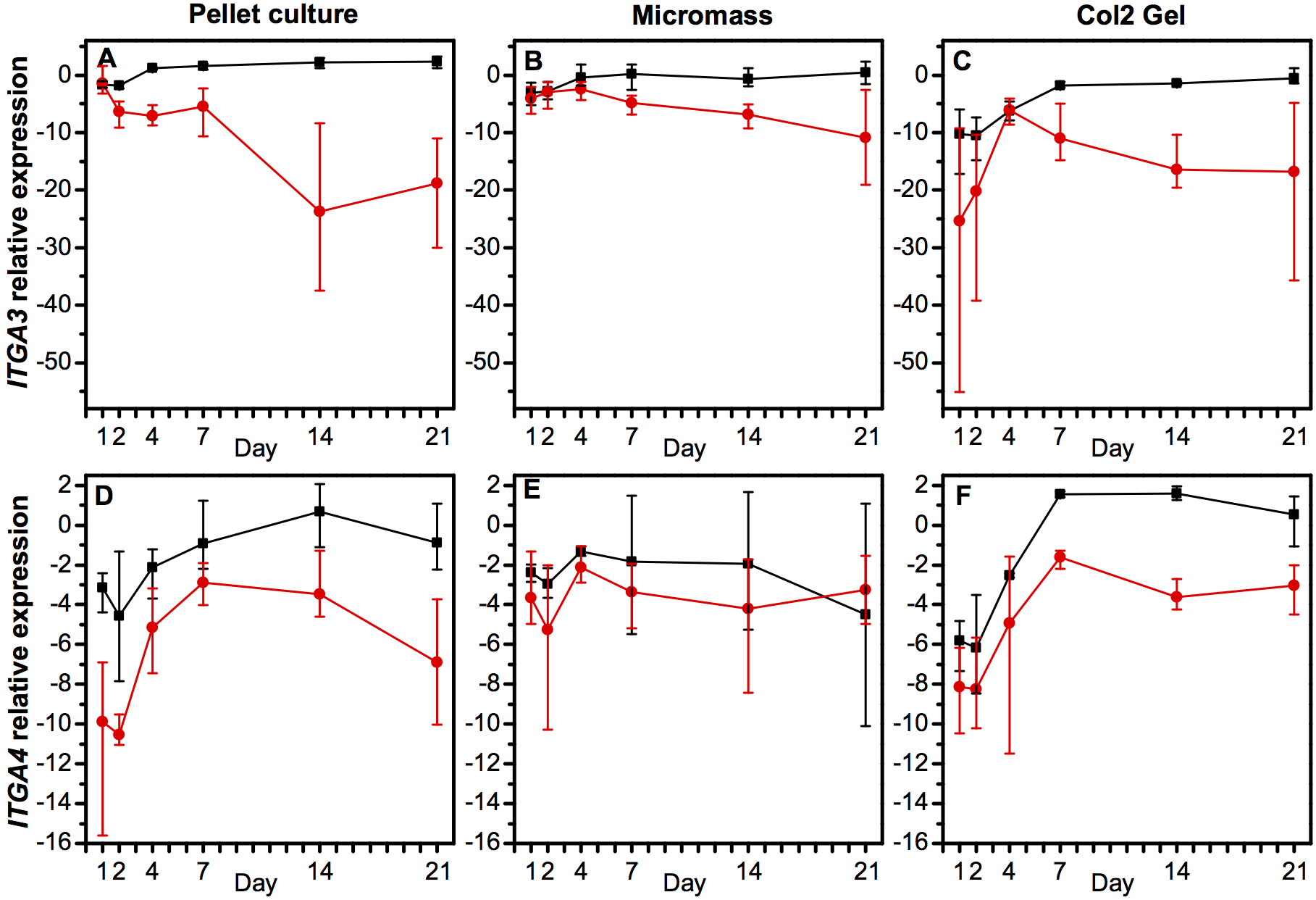

Supplement: Figure S3 — Quantitative PCR established mRNA expression of integrin subunits ITGA3 (A-C) and ITGA4 (D-F) in hMSCs cultured in three different chondrogenesis models (pellet culture, micromass culture, or a type II collagen hydrogel) in either growth (black squares) or chondrogenic (red circles) medium over a time-course of 21 days. Each point represents mean expression relative to GAPDH of N=3 independent experiments, and error bars represent the range of values. Statistical significance is in Figures S12-S14. (TIFF) [file pone.0082035.s003.tiff]

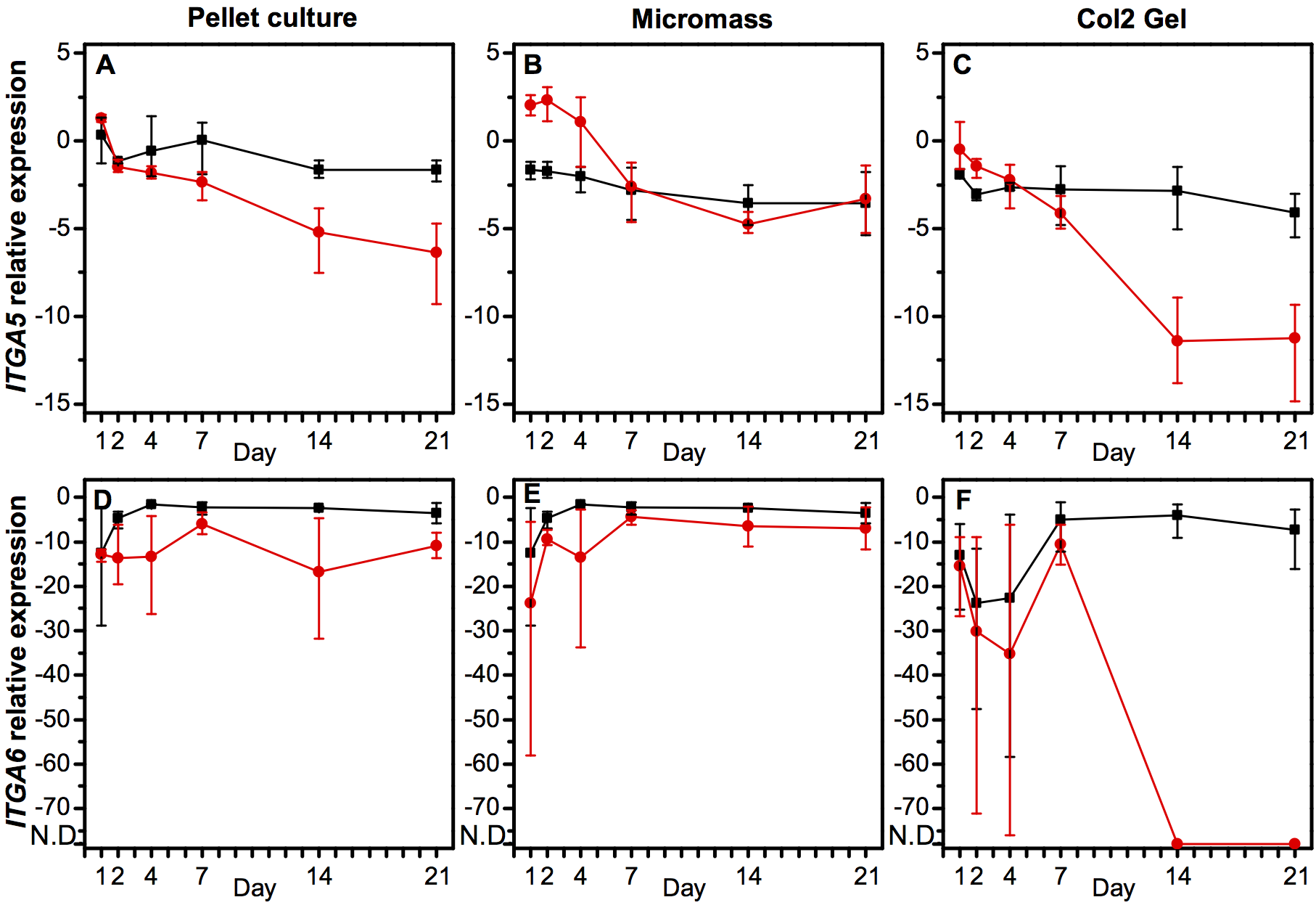

Supplement: Figure S4 — Quantitative PCR established mRNA expression of integrin subunits ITGA5 (A-C) and ITGA6 (D-F) in hMSCs cultured in three different chondrogenesis models (pellet culture, micromass culture, or a type II collagen hydrogel) in either growth (black squares) or chondrogenic (red circles) medium over a time-course of 21 days. Each point represents mean expression relative to GAPDH of N=3 independent experiments, and error bars represent the range of values. Statistical significance is in Figures S12-S14. (TIFF) [file pone.0082035.s004.tiff]

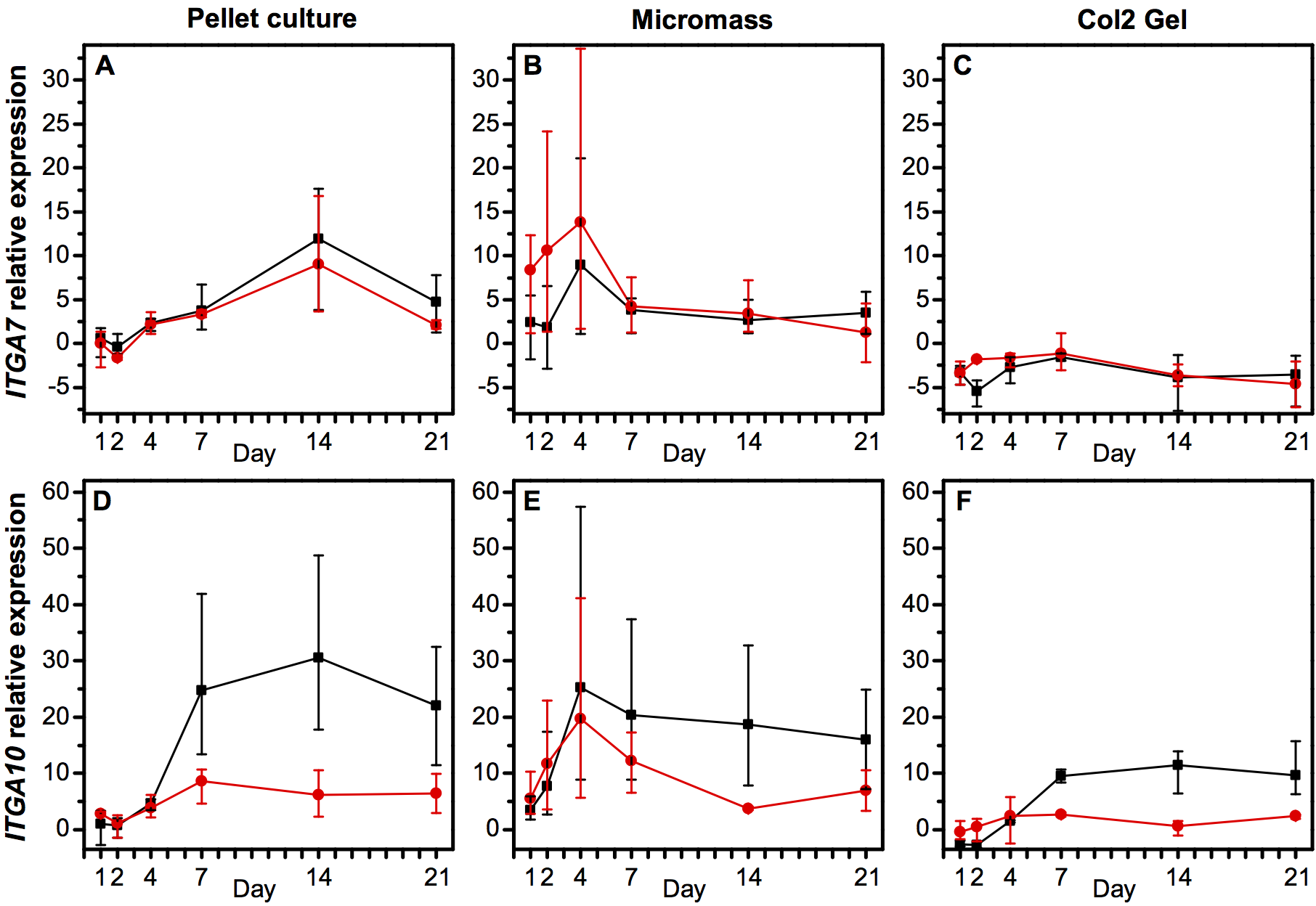

Supplement: Figure S5 — Quantitative PCR established mRNA expression of integrin subunits ITGA7 (A-C) and ITGA10 (D-F) in hMSCs cultured in three different chondrogenesis models (pellet culture, micromass culture, or a type II collagen hydrogel) in either growth (black squares) or chondrogenic (red circles) medium over a time-course of 21 days. Each point represents mean expression relative to GAPDH of N=3 independent experiments, and error bars represent the range of values. Statistical significance is in Figures S12-S14. (TIFF) [file pone.0082035.s005.tiff]

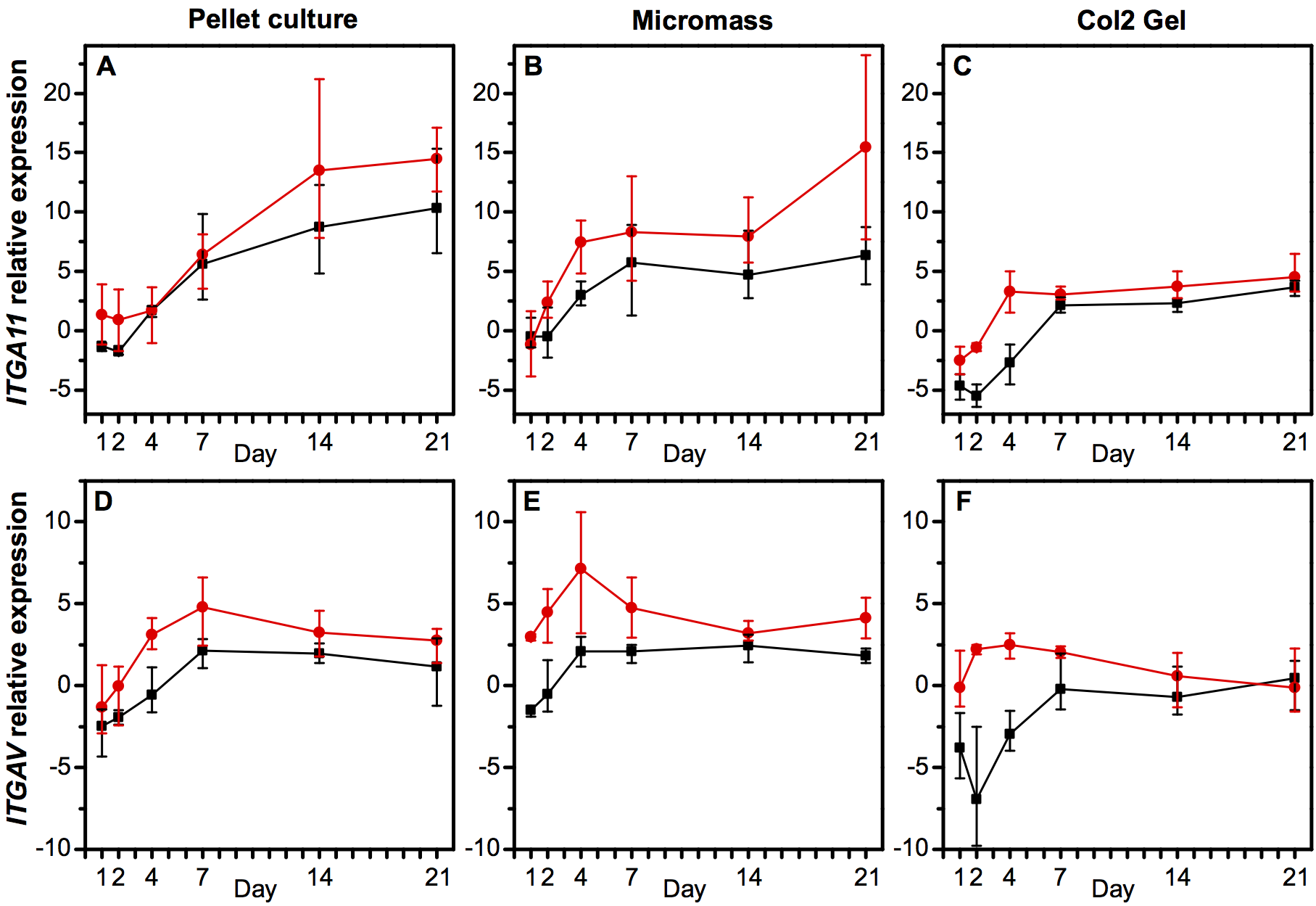

Supplement: Figure S6 — Quantitative PCR established mRNA expression of integrin subunits ITGA11 (A-C) and ITGAV (D-F) in hMSCs cultured in three different chondrogenesis models (pellet culture, micromass culture, or a type II collagen hydrogel) in either growth (black squares) or chondrogenic (red circles) medium over a time-course of 21 days. Each point represents mean expression relative to GAPDH of N=3 independent experiments, and error bars represent the range of values. Statistical significance is in Figures S12-S14. (TIFF) [file pone.0082035.s006.tiff]

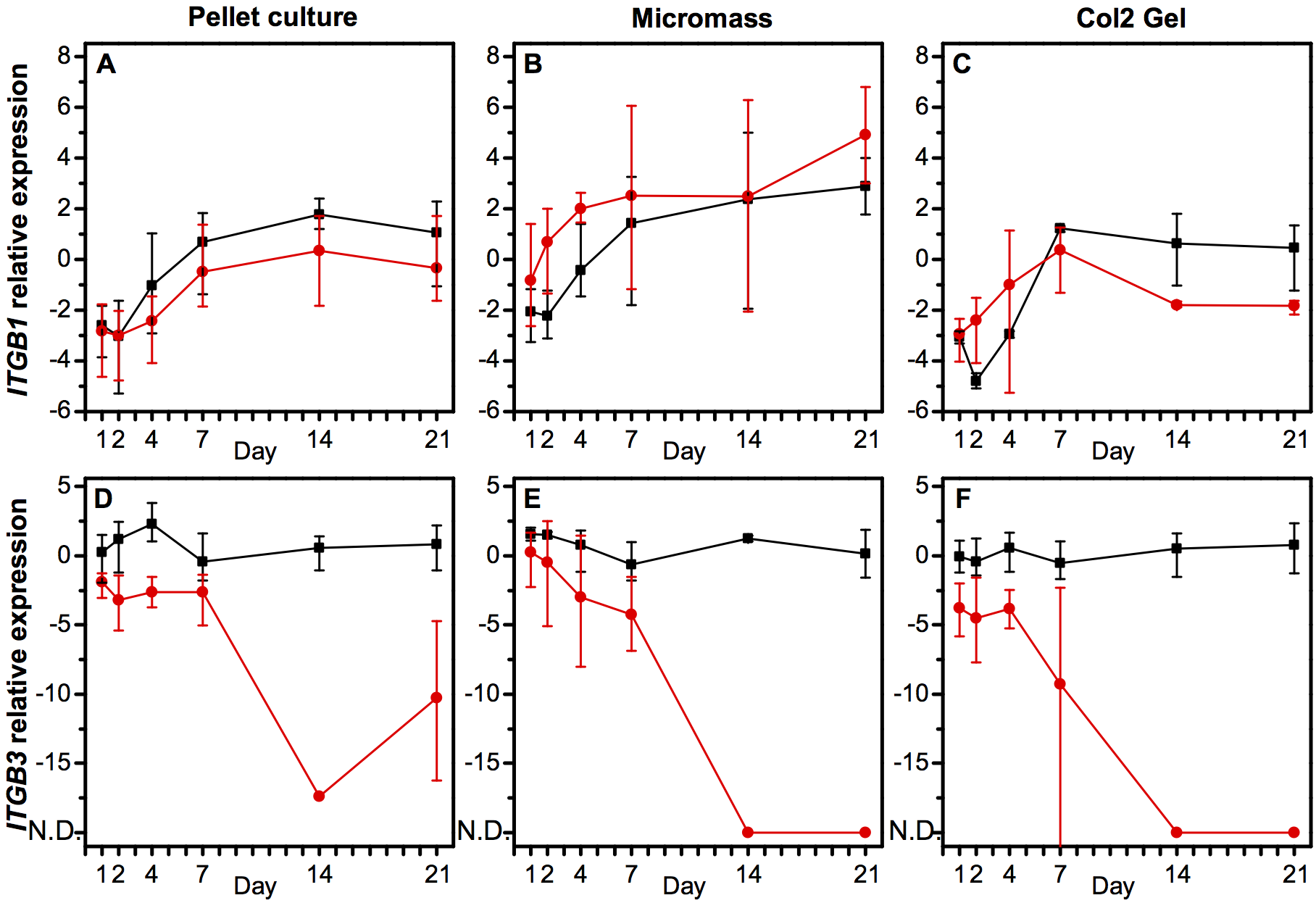

Supplement: Figure S7 — Quantitative PCR established mRNA expression of integrin subunits ITGB1 (A-C) and ITGB3 (D-F) in hMSCs cultured in three different chondrogenesis models (pellet culture, micromass culture, or a type II collagen hydrogel) in either growth (black squares) or chondrogenic (red circles) medium over a time-course of 21 days. Each point represents mean expression relative to GAPDH of N=3 independent experiments, and error bars represent the range of values. Statistical significance is in Figures S12-S14. (TIFF) [file pone.0082035.s007.tiff]

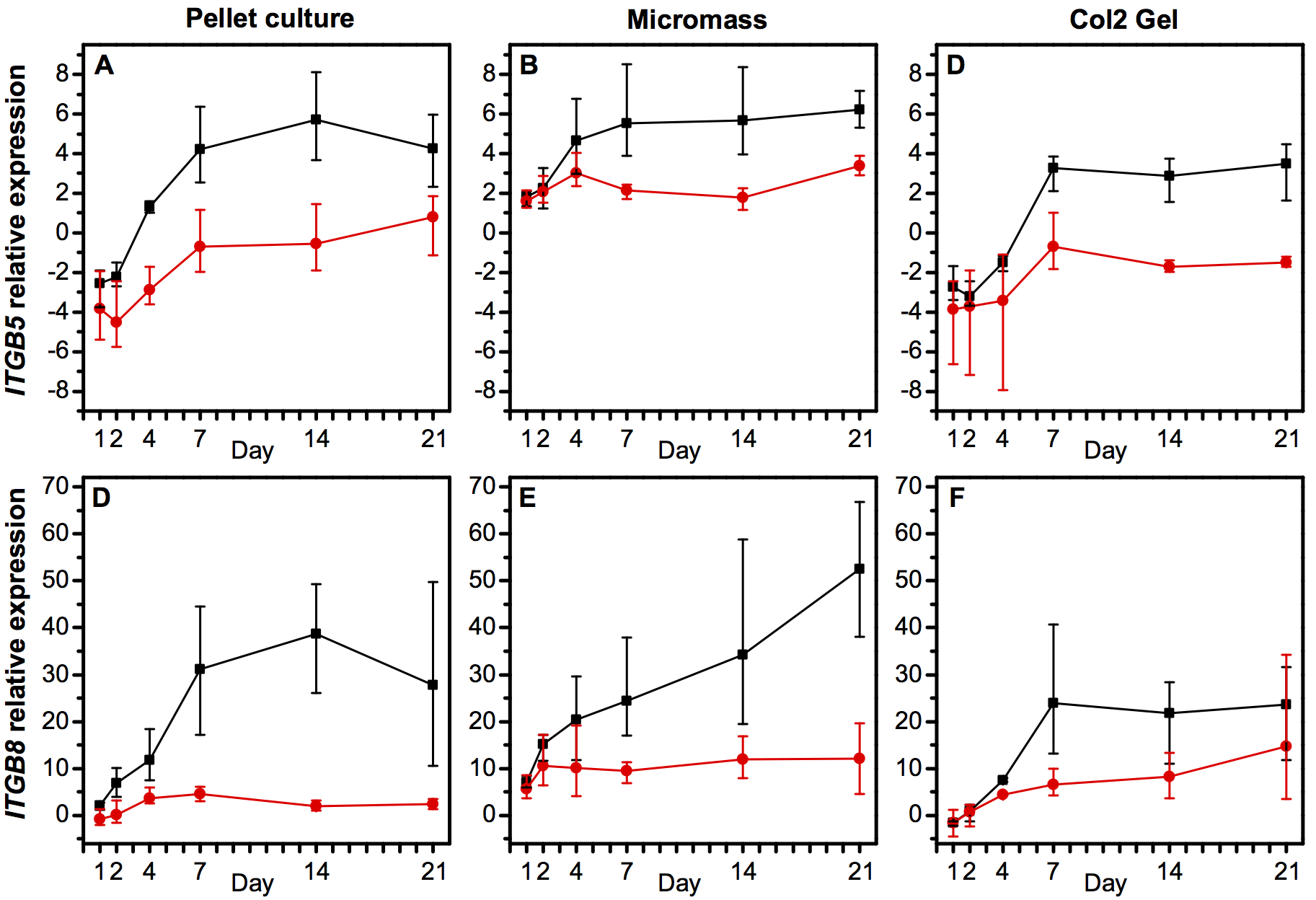

Supplement: Figure S8 — Quantitative PCR established mRNA expression of integrin subunits ITGB5 (A-C) and ITGB8 (D-F) in hMSCs cultured in three different chondrogenesis models (pellet culture, micromass culture, or a type II collagen hydrogel) in either growth (black squares) or chondrogenic (red circles) medium over a time-course of 21 days. Each point represents mean expression relative to GAPDH of N=3 independent experiments, and error bars represent the range of values. Statistical significance is in Figures S12-S14. (TIFF) [file pone.0082035.s008.tiff]

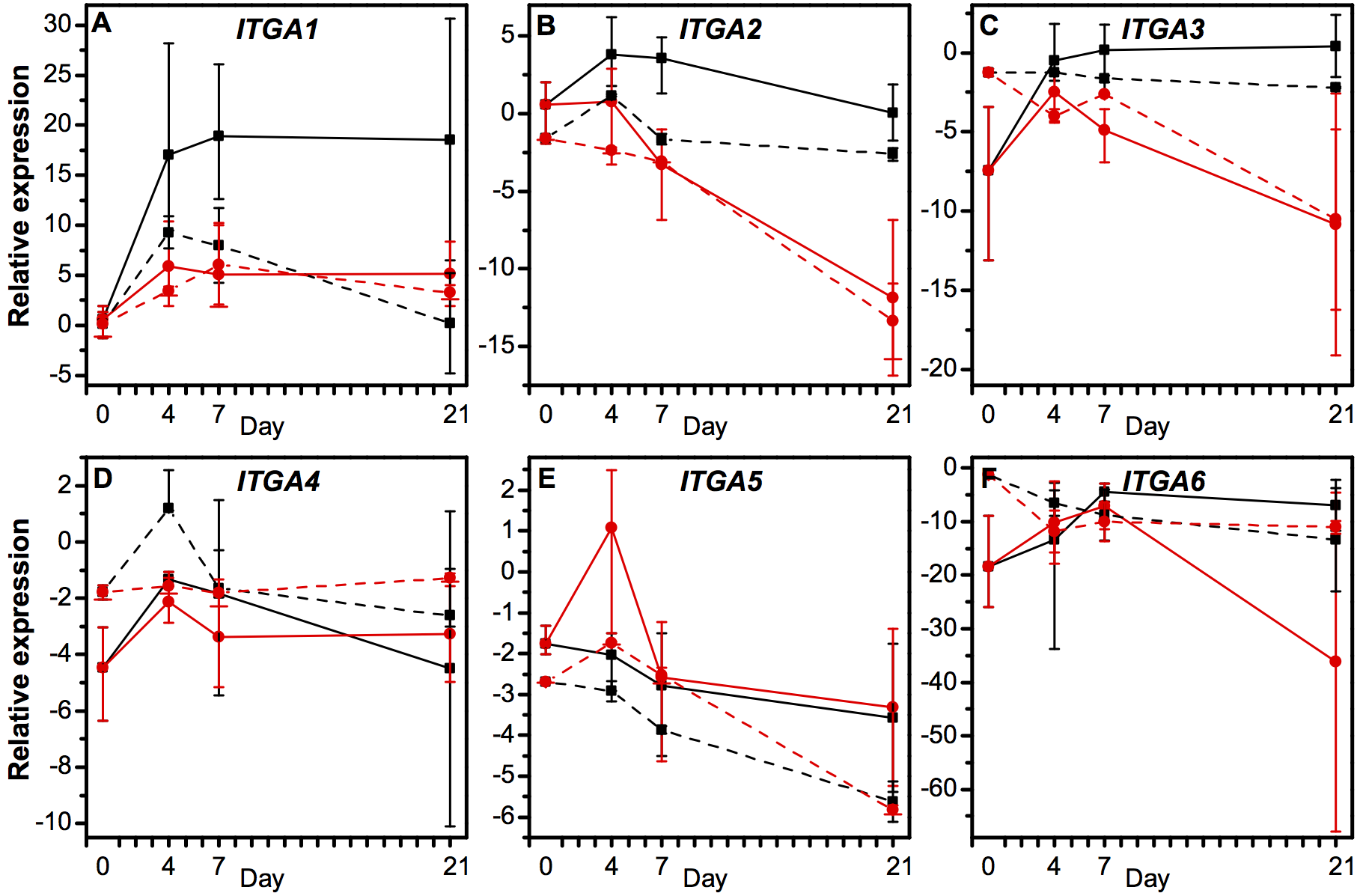

Supplement: Figure S9 — Quantitative PCR established mRNA expression of integrin subunits in hMSCs (wildtype: solid lines, ITGB8 knockdown: dashed lines) cultured in a micromass in either growth (black squares) or chondrogenic (red circles) medium over a time-course of 21 days. A) ITGA1, B) ITGA2, C) ITGA3, D) ITGA4, E) ITGA5, and F) ITGA6 were unaffected by the ITGB8 knockdown after 21 days. Each point represents mean expression relative to GAPDH and to hMSCs on tissue culture plastic in growth medium of N=2-3 independent experiments, and error bars represent the range of values. Statistical significance is in Figure S17. (TIFF) [file pone.0082035.s009.tiff]

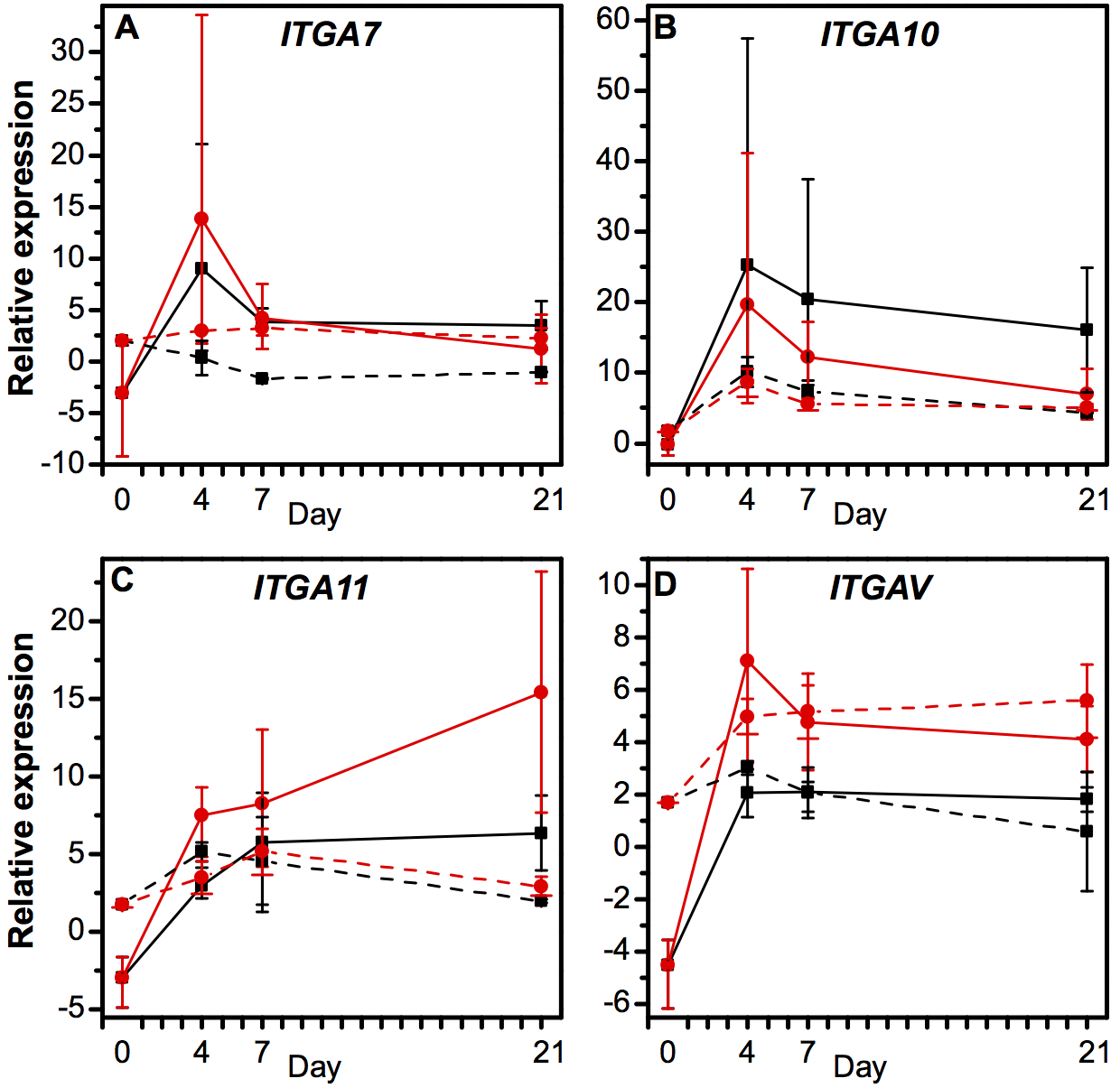

Supplement: Figure S10 — Quantitative PCR established mRNA expression of integrin subunits in hMSCs (wildtype: solid lines, ITGB8 knockdown: dashed lines) cultured in a micromass in either growth (black squares) or chondrogenic (red circles) medium over a time-course of 21 days. A) ITGA7, B) ITGA10, C) ITGA11, and D) ITGAV were unaffected by the ITGB8 knockdown after 21 days. Each point represents mean expression relative to GAPDH and to hMSCs on tissue culture plastic in growth medium of N=2-3 independent experiments, and error bars represent the range of values. Statistical significance is in Figure S17. (TIFF) [file pone.0082035.s010.tiff]

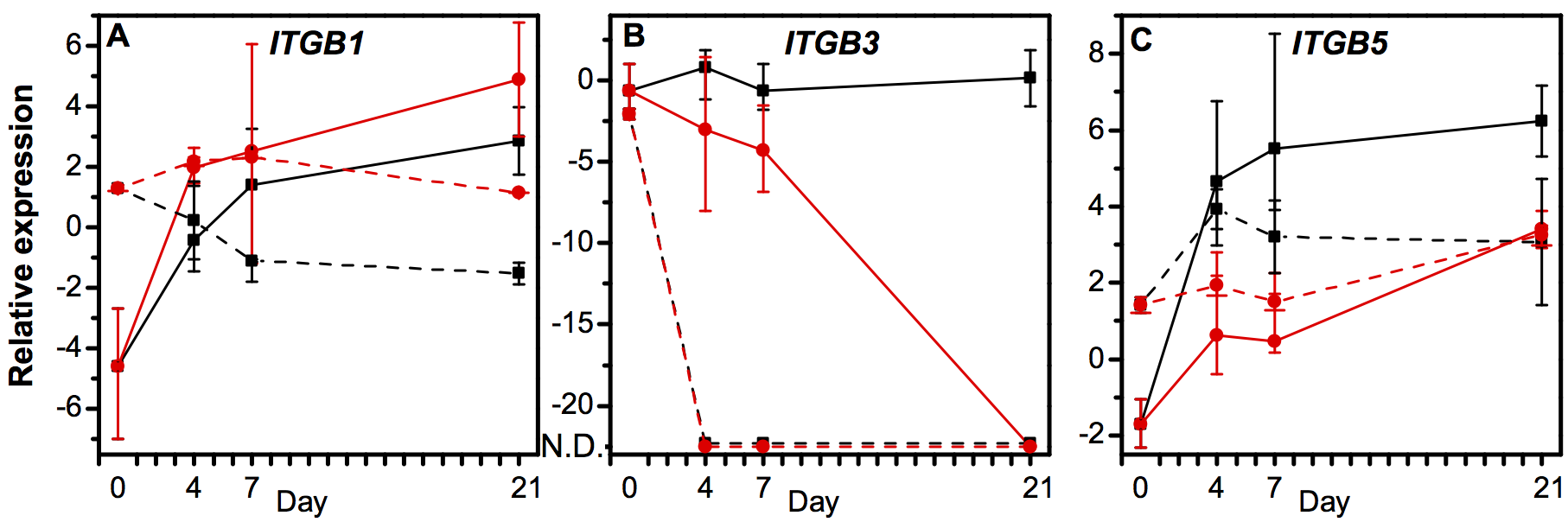

Supplement: Figure S11 — Quantitative PCR established mRNA expression of integrin subunits in hMSCs (wildtype: solid lines, ITGB8 knockdown: dashed lines) cultured in a micromass in either growth (black squares) or chondrogenic (red circles) medium over a time-course of 21 days. A) ITGB1 was unaffected, B) ITGB3 was undetectable, and C) ITGB5 was unaffected by the ITGB8 knockdown after 21 days. Each point represents mean expression relative to GAPDH and to hMSCs on tissue culture plastic in growth medium of N=2-3 independent experiments, and error bars represent the range of values. N.D.: not detected. Statistical significance is in Figure S17. (TIFF) [file pone.0082035.s011.tiff]

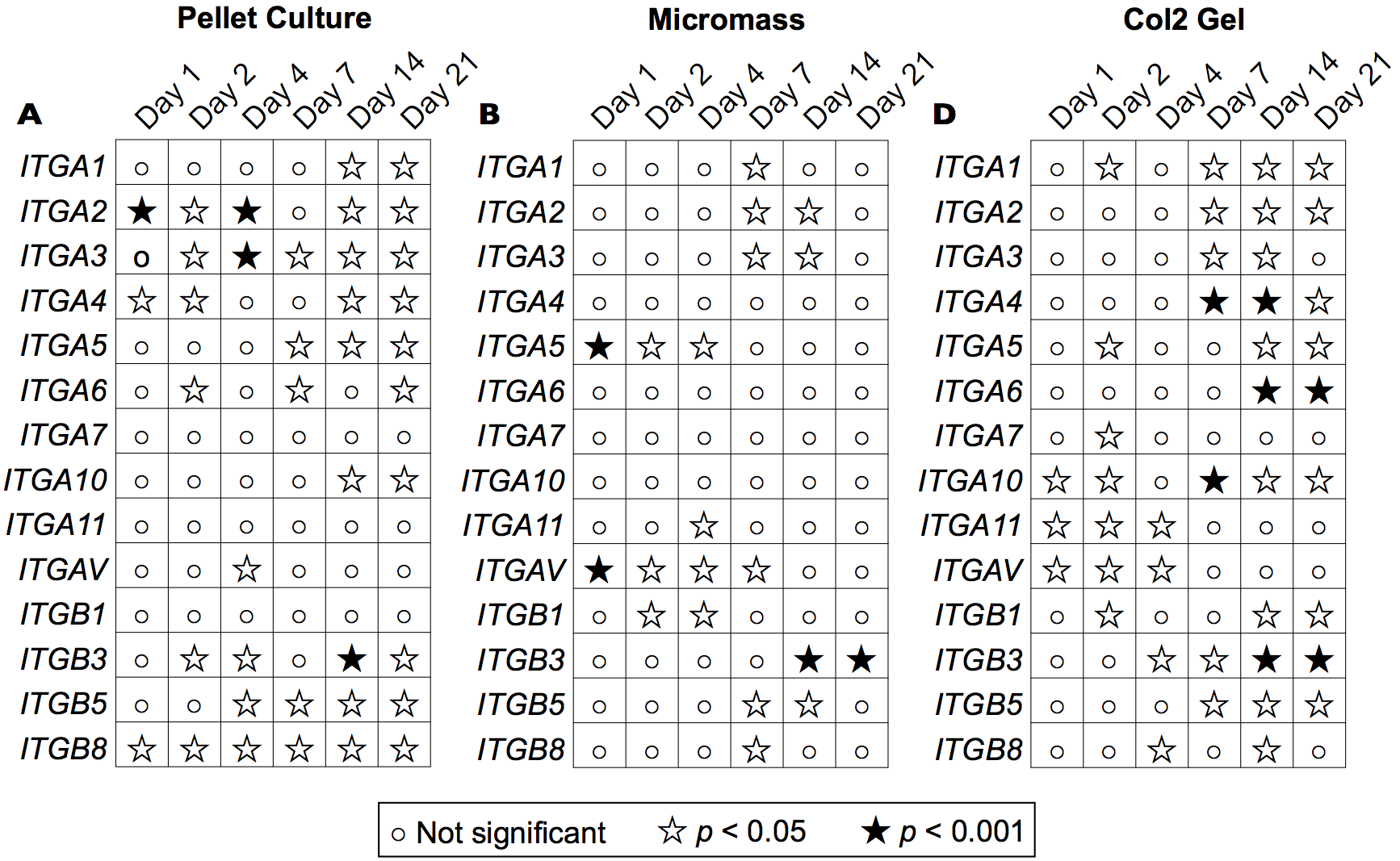

Supplement: Figure S12 — The statistical significance of the effect of the medium composition on integrin expression from Figure 5. Student's t-test was used to determine p-values to indicate whether there was a statistically significant difference between cells cultured in growth or chondrogenic medium at each time-point in each of the three chondrogenesis models, A) pellet culture, B) micromass, or C) type II collagen hydrogel. N=3 independent experiments in technical triplicates. (TIFF) [file pone.0082035.s012.tiff]

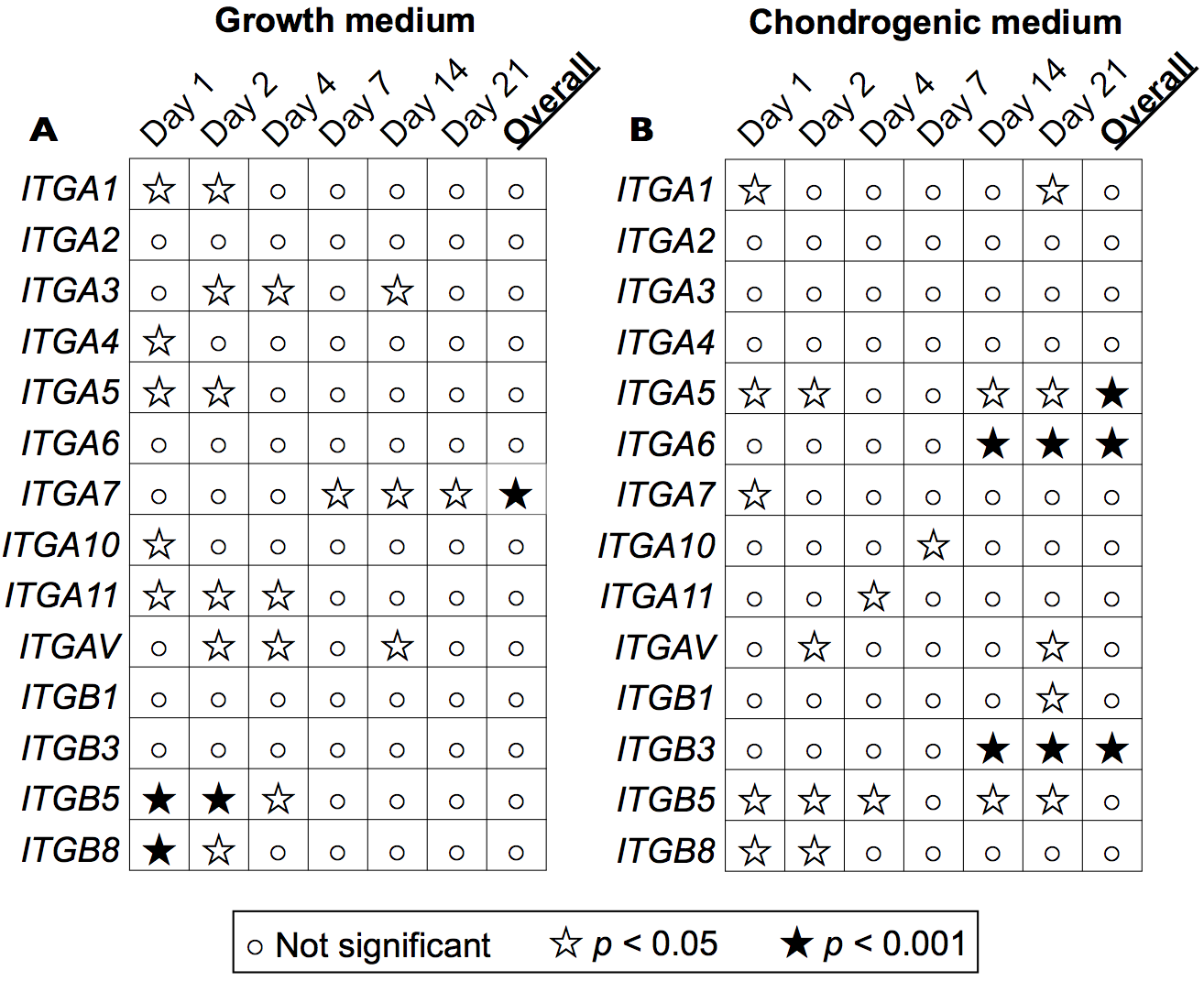

Supplement: Figure S13 — The statistical significance of the effect of the chondrogenesis model on integrin expression from Figure 5. A two-way ANOVA was used to determine p-values to indicate whether there was a statistically significant difference between cells cultured in pellet culture, micromass, or a type II collagen hydrogel in either A) growth medium, or B) chondrogenic medium. N=3 independent experiments in technical triplicates. (TIFF) [file pone.0082035.s013.tiff]

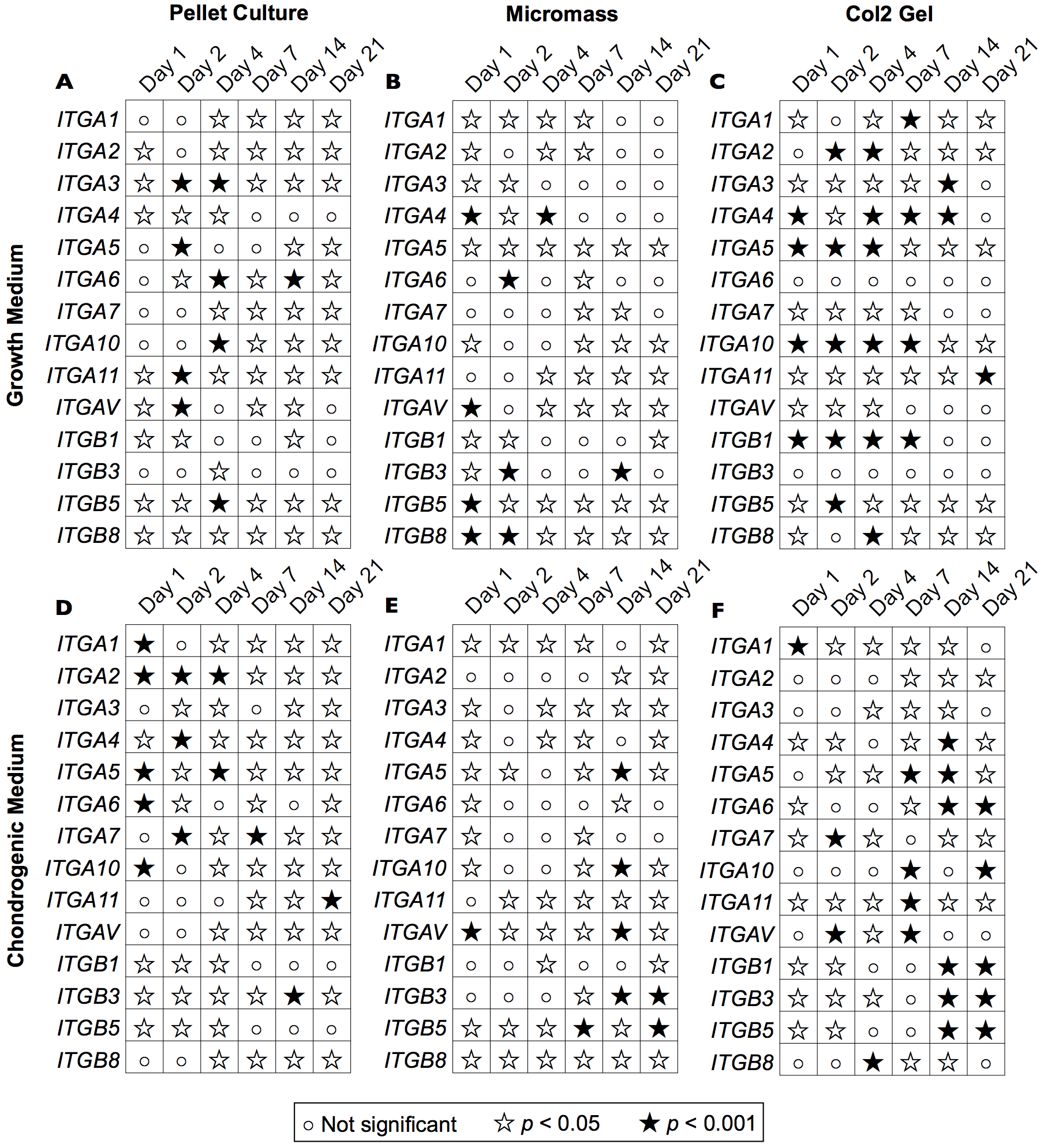

Supplement: Figure S14 — The statistical significance of the effect of the time-course on integrin expression from Figure 5. Student's t-test was used to determine whether there was a statistically significant difference in integrin expression at each time-point in each of the three chondrogenesis models: A and D) pellet culture, B and E) micromass, or C and F) type II collagen hydrogel, when compared to hMSCs at day 0 in growth medium. N=3 independent experiments in technical triplicates. (TIFF) [file pone.0082035.s014.tiff]

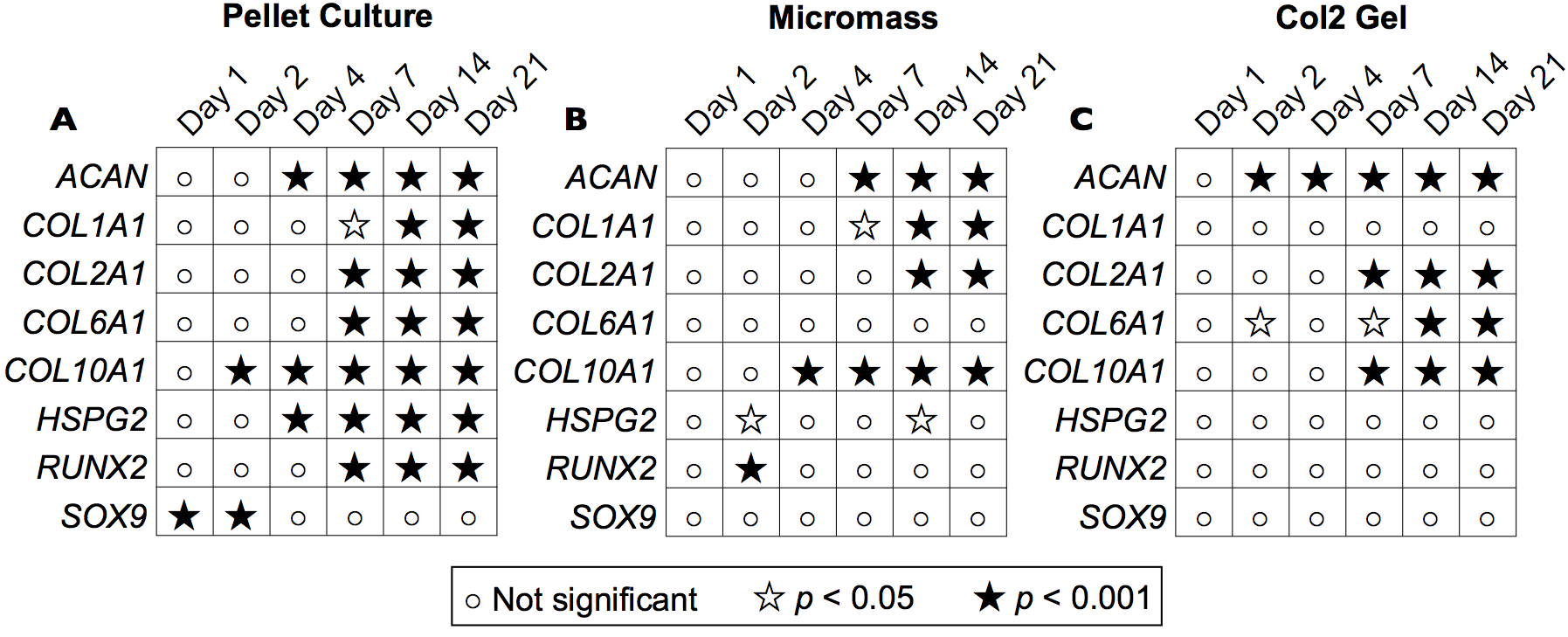

Supplement: Figure S15 — The statistical significance of the effect of the medium composition on phenotype marker expression from Figures 2-3. Student's t-test was used to determine p-values to indicate whether there was a statistically significant difference between cells cultured in growth or chondrogenic medium at each time-point in each of the three chondrogenesis models, A) pellet culture, B) micromass, or C) type II collagen hydrogel. N=3 independent experiments in technical triplicates. (TIFF) [file pone.0082035.s015.tiff]

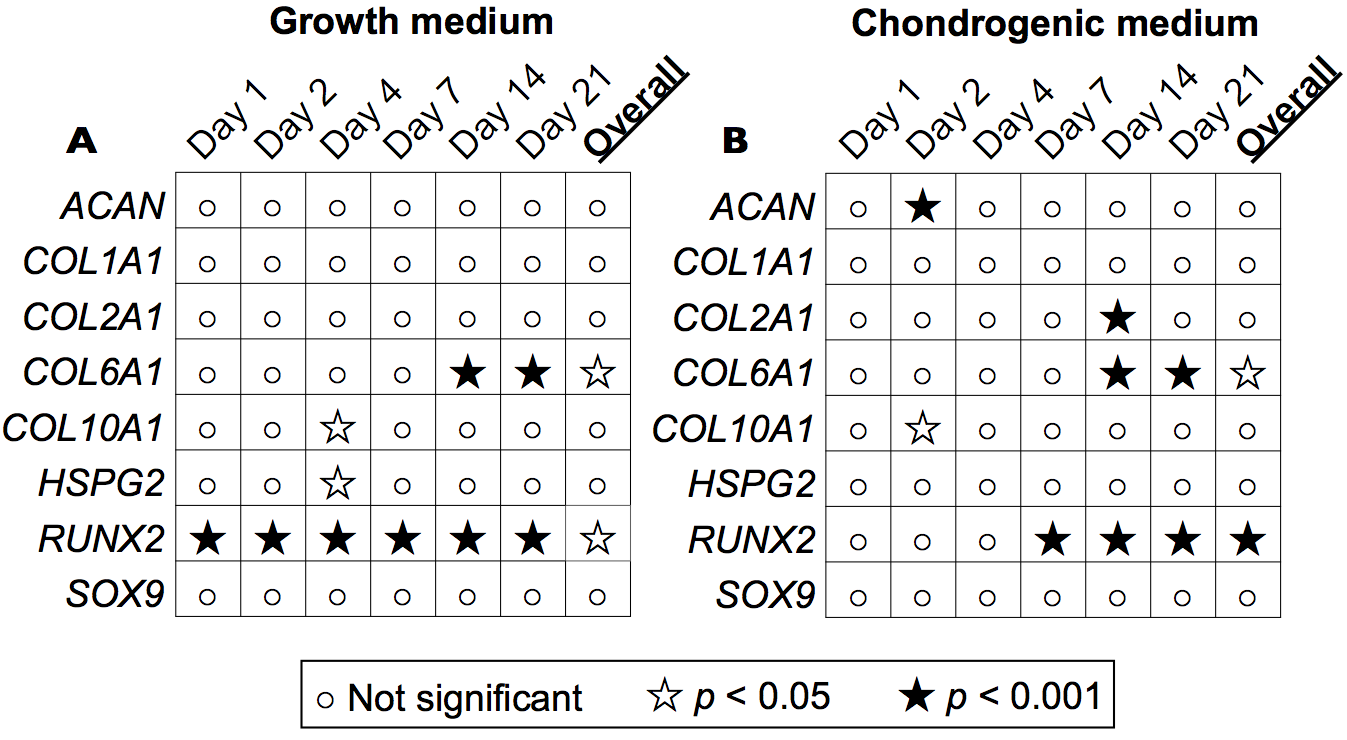

Supplement: Figure S16 — The statistical significance of the effect of the chondrogenesis model on phenotype marker expression from Figures 2-3. A two-way ANOVA was used to determine p-values to indicate whether there was a statistically significant difference between cells cultured in pellet culture, micromass, or a type II collagen hydrogel in either A) growth medium, or B) chondrogenic medium. N=3 independent experiments in technical triplicates. (TIFF) [file pone.0082035.s016.tiff]

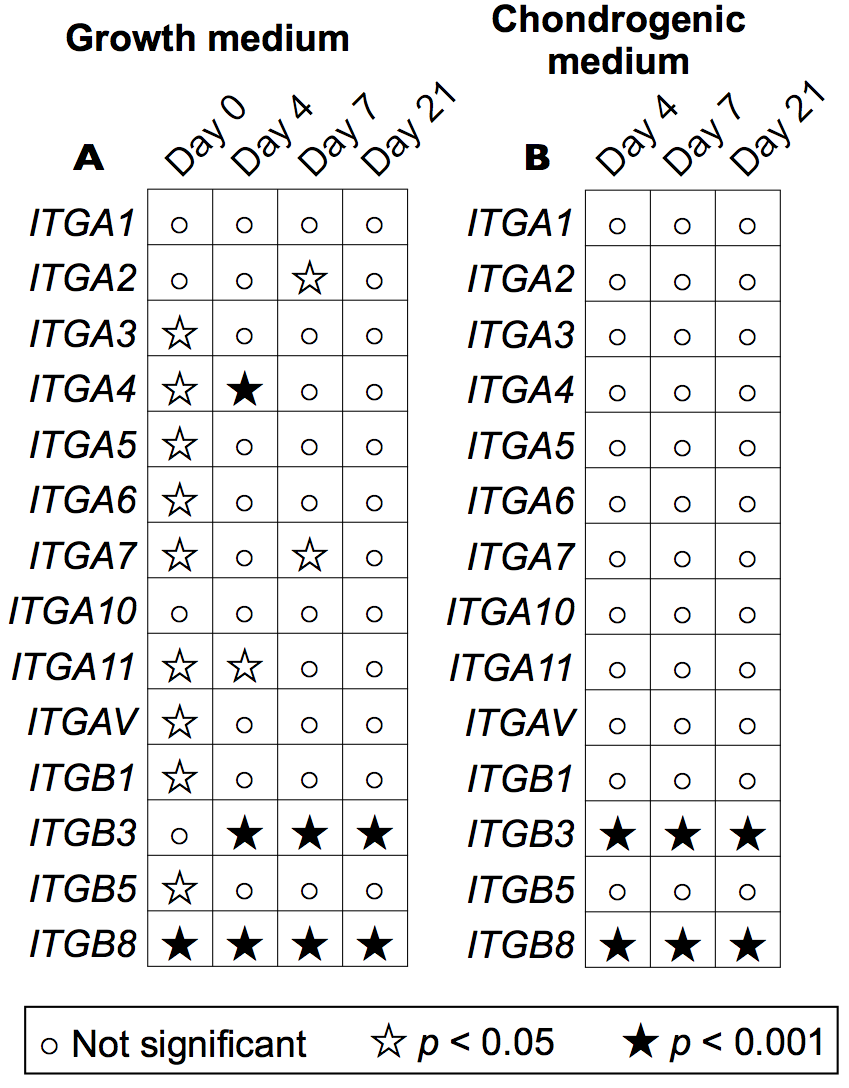

Supplement: Figure S17 — The statistical significance of the effect of the knockdown of ITGB8 on integrin expression. Student's t-test was used to determine p-values to indicate whether there was a statistically significant difference in transcript expression between the knockdown and the wildtype in either A) growth medium, or B) chondrogenic medium. N=2-3 independent experiments in technical triplicates. (TIFF) [file pone.0082035.s017.tiff]

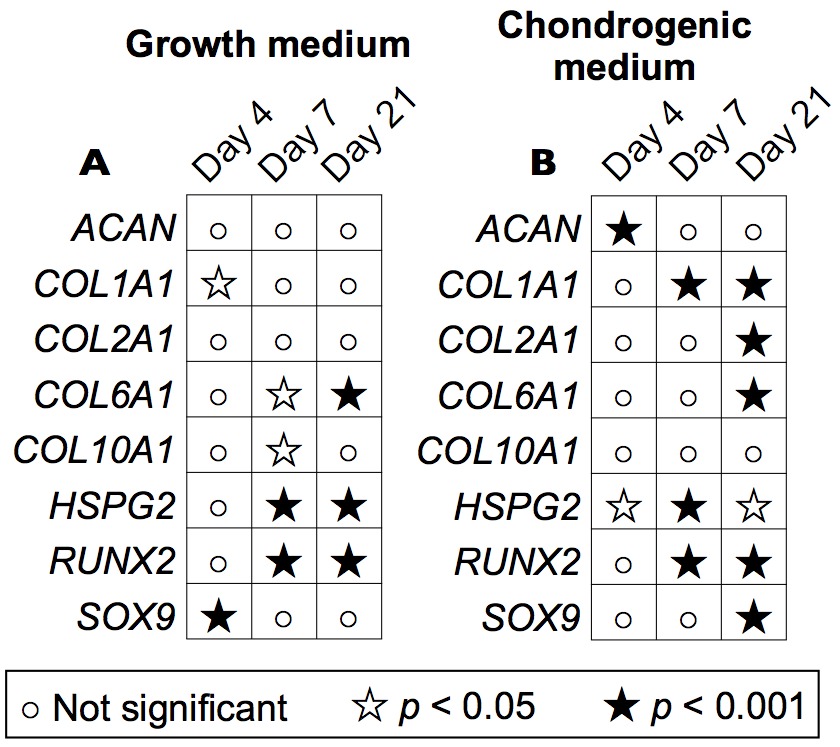

Supplement: Figure S18 — The statistical significance of the effect of the knockdown of ITGB8 on phenotype marker expression from Figure 6. Student's t-test was used to determine p-values to indicate whether there was a statistically significant difference in transcript expression between the knockdown and the wildtype in either A) growth medium, or B) chondrogenic medium. N=2-3 independent experiments in technical triplicates. (TIFF) [file pone.0082035.s018.tiff]

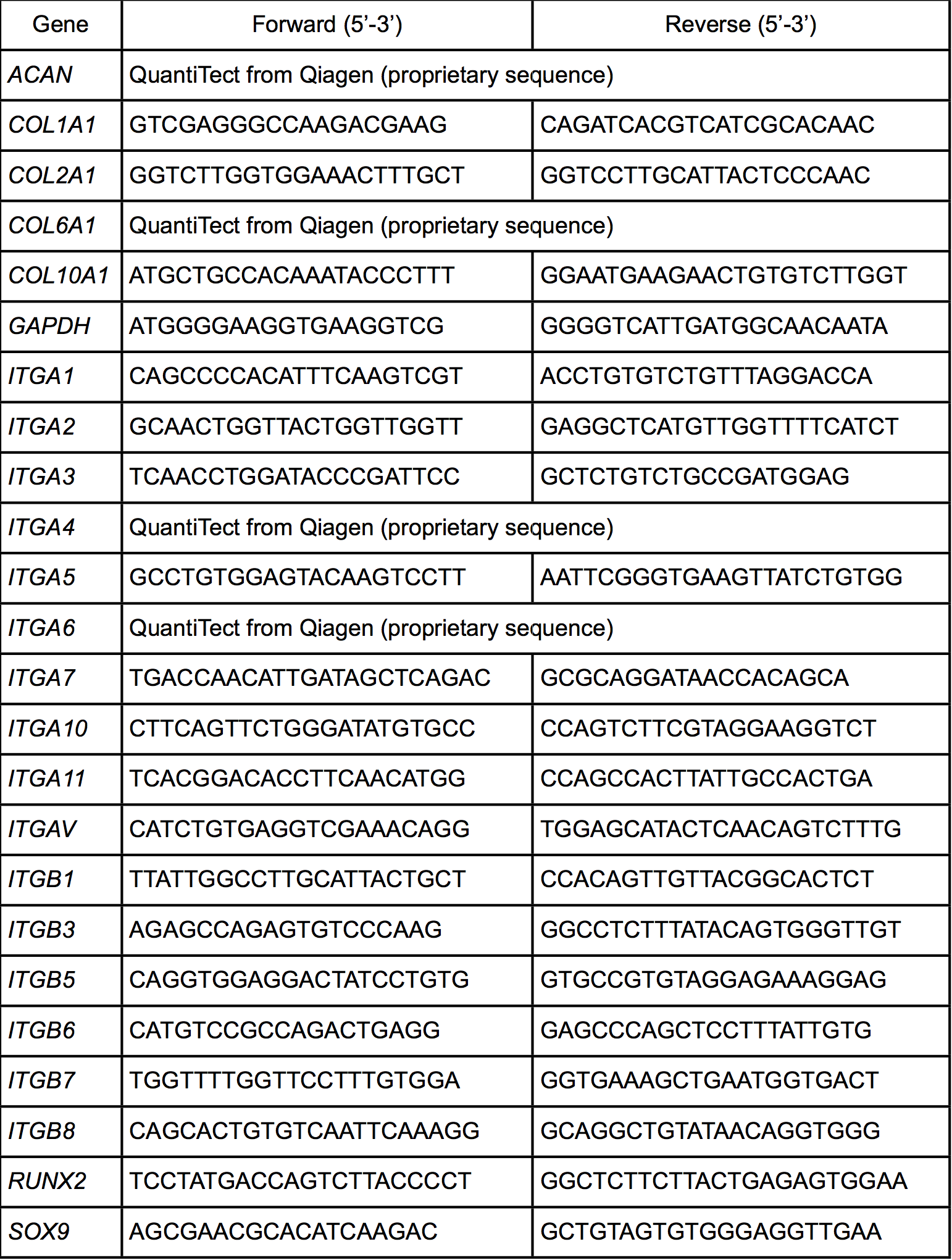

Supplement: Table S1 — Quantitative PCR primer sequences used in this study. (TIFF) [file pone.0082035.s019.tiff]
